# Characterization and Expression Patterns of microRNAs Involved in Rice Grain Filling

**DOI:** 10.1371/journal.pone.0054148

**Published:** 2013-01-24

**Authors:** Ting Peng, Hongzheng Sun, Yanxiu Du, Jing Zhang, Junzhou Li, Yanxia Liu, Yafan Zhao, Quanzhi Zhao

**Affiliations:** Research Center for Rice Engineering of Henan Agricultural University and Key Laboratory of Physiology, Ecology and Genetic Improvement of Food Crops in Henan Province, Zhengzhou, China; East Carolina University, United States of America

## Abstract

MicroRNAs (miRNAs) are upstream gene regulators of plant development and hormone homeostasis through their directed cleavage or translational repression of the target mRNAs, which may play crucial roles in rice grain filling and determining the final grain weight and yield. In this study, high-throughput sequencing was performed to survey the dynamic expressions of miRNAs and their corresponding target genes at five distinct developmental stages of grain filling. In total, 445 known miRNAs and 45 novel miRNAs were detected with most of them expressed in a developmental stage dependent manner, and the majority of known miRNAs, which increased gradually with rice grain filling, showed negatively related to the grain filling rate. Detailed expressional comparisons revealed a clear negative correlation between most miRNAs and their target genes. It was found that specific miRNA cohorts are expressed in a developmental stage dependent manner during grain filling and the known functions of these miRNAs are involved in plant hormone homeostasis and starch accumulation, indicating that the expression dynamics of these miRNAs might play key roles in regulating rice grain filling.

## Introduction

As one of the most important cereal crops worldwide, rice (*Oryza sativa*) provides over 21% of the calorific needs of the world's population and up to 76% of the calorific intake of the population of South East Asia [1]. The yield of rice needs to be increased rapidly to meet the expanding food demands of the rapidly growing world population and economic development. As we all know, four components contribute to potential rice grain yield, that is, grain weight, grain number per panicle, panicle number per plant, and the ratio of filled grains [2]. It has been documented that grain weight is largely determined by the grain filling [3]. Therefore, research on the grain filling in rice is helpful to enhance the grain weight, hence increasing the grain yield per plant.

Rice grain filling is a process of starch accumulation, because starch contributes 80–90% of the final dry weight of brown rice [4]. The process of grain filling is a complex series of events, including relocation of carbohydrates synthesized by photosynthetic organs and biosynthesis of starch from sucrose in the developing endosperm. It has been reported that several genes, such as GIF1 [3], GS5 [5] and starch synthesis related genes [4] play crucial roles on rice grain filling. The activity of key enzymes, such as, sucrose synthase (SUS), adenosine diphosphate-glucose pyrophosphorylase (AGPase), starch synthase (SS), and starch branching enzyme (SBE) were positively correlated with rice grain filling rate [6]. Higher level of plant hormones such as ABA, cytokinins, IAA, and brassinosteroids, were significantly and closely related to a higher grain filling rate and starch accumulation [7]–[10], whereas a higher ethylene level in developing seeds correlates negatively with starch metabolism related enzyme activities and always leads to poor grain filling [7]. However, roles of upstream regulators of these coding genes, such as miRNAs, have not yet been fully studied.

MiRNAs are a class of 20 to 24 nucleotide endogenous small RNAs which can regulate gene expression by cleavage or translational repression of the target gene transcripts [11]. They are proved to play important roles in regulating plant development [12]–[14], hormone homeostasis [15], [16], and stress responses [17], [18]. With the development of sequencing technology, deep sequencing has been widely used to identify species or tissue specific miRNAs [19] and to compare the expression of miRNAs between different development stages, treatments or genotypes [20]–[24]. In recent years, miRNAs in rice seed development were identified and studied in several reports [20], [24], [25]. In rice grains, most miRNAs were expressed higher in 6 to 10 days after fertilization compared with the earlier stage (1 to 5 days after fertilization) [20]. Most recently study showed that approximately half of the detected miRNAs were up-regulated, whereas the remainder were down-regulated with the development of rice grains in Indica rice filling phase (6–20 DAF) [24]. A rice miRNA, (miR167), may play a role in rice grain development through an auxin-miR167-ARF8-OsGH3.2 pathway, while other miRNAs such as miR397, miR398, miR408, and miR528 may have important adjusting effects on rice grain development through investigating the expression of miRNAs in the mixture of immature seeds at 3, 6, 9 and 12 days after anthesis [25]. However, all of these studies focused on the prometaphase of rice grain filling. In rice seeds, starch begins to accumulate from 4 days after flowering (DAF) [26], and gets maturation around 35DAF. To the best of our knowledge, the expression patterns of miRNAs and their regulate roles in the whole rice grain filling stage have not been fully studied.

In order to investigate the dynamic expression profile of miRNAs during rice grain filling in japonica rice, the small RNA populations were investigated at 10DAF, 15DAF, 21DAF, 27DAF and 35DAF by high-throughput sequencing technology. In parallel, digital gene expression profiling (DGE) was carried out to compare the expression patterns between miRNAs and their corresponding target genes. As a result, 445 known miRNAs, and 45 novel miRNAs were detected. Furthermore, the potential roles of miRNAs during grain filling were discussed, combined with DGE results, the result indicated that the predicted targets of those miRNAs are involved in many metabolic processes, such as hormone homeostasis and starch accumulations.

## Materials and Methods

### Plant materials, and cultivation

Oryza sativa spp. japonica cv. Xinfeng 2 were planted from the same seed lot and grown at a research farm of Henan Agricultural University, Henan Province, China (34°53′N, 113°35′E, 94 m altitude) under non-stress conditions during the rice-growing season. The superior spikelets (n = 35), located at the top of the panicle as experimental materials as Xu et al [26], sampled at 5 d intervals from fertilization to 45 DAF as the method used by Ishimaru et al [27], were used to measure dry grain weight at each sampling time. The grains were separated from the panicle, fast dried at 105°C for 30 min, and then kept at 80°C for a complete dryness until reaching a constant weight. The processes of grain filling were fitted by Logistic growth equation:
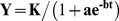
(1)


The grain filling rate (V) was calculated as the derivative of Equation 1:
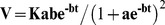
(2)where Y is the mean weight per grain (mg), t is number of days after flowering (DAF), K, a and b are coefficients determined by regression.

### RNA isolation, library construction, Solexa sequencing and analysis of the deep sequencing data

Total RNA were isolated from dehusked seeds at 10DAF, 15DAF, 21DAF, 27DAF, and 35DAF using TRIzol reagent (Invitrogen, Carlsbad, CA, USA) according to the manufacturer's instructions, then RNase-free DNase I was used to remove the residual DNA for 30 min at 37°C separately. The small RNAs sized at 18–30 nt were gel purified on a 15% polyacrylamide denaturing gel from the total RNA of the five samples. Then the isolated small RNAs were ligated to RNA–DNA chimeric oligo nucleotide adaptors, reverse transcribed, and amplified with 15 cycles of PCR to produce sequencing libraries. The final PCR product was purified and sequenced by using Solexa sequencing technology at Beijing Genomics Institute (Shenzhen, China) according to the manufacturer's protocols, respectively.

After removing the adapter sequences and low quality sequences of the deep-sequencing raw data, the resulting unique small RNAs from the five libraries were aligned to the rice genome using SOAP [28]. Whole matching sequences were classified into tRNA, rRNA, small nucleolar RNA (snoRNA), and small nuclear RNA (snRNA) gene derived small RNAs by comparison with the NCBI nucleotide database and Rfam RNA family database [29]. Small RNAs mapped to these non-coding RNAs were removed from the dataset. Then the remainder small RNAs were aligned to miRNA precursor sequences of rice miRNA database available in miRBase (miRBase release 17) to obtain the known miRNAs. And the sequences located at the position ±2 nt of the precursors were also included as mature miRNAs. Small RNAs positioned at repeat loci and aligned to exon and intron of mRNA were also identified and annotated. To ensure every unique small RNAs mapped to only one annotation, we follow the following priority rule: rRNAetc (in which Genbank > Rfam) > known miRNA > repeat > exon > intron. Finally, to compare the expression patterns of these miRNAs during grain filling, the abundance of each miRNAs in the five libraries were normalized to transcripts per million (TPM) as we previously described [30].

### Identification of novel miRNAs

After mapping the clean reads to NCBI nucleotide database, Rfam RNA family database, and miRBase, the unannotated small RNAs were subjected to ‘MIREAP’ (https://sourceforge.net/projects/mireap/) to identify the novel miRNA candidates under the following parameters: minimal candidate sequence length (18), maximal candidate sequence length (25), maximal free energy allowed for a candidate miRNA precursor (−18 kcal/mol), maximal space between candidate miRNA and miRNA* (300), minimal base pairs of candidate miRNA and miRNA* (16), maximal bulge of candidate miRNA and miRNA* (4), maximal asymmetry of candidate miRNA/miRNA* duplex (4), flank sequence length of candidate miRNA precursor (20). Furthermore, only the candidate satisfied the following criteria was considered as a novel miRNA: the candidate has the presence of their corresponding miRNA* sequences, a strong indication of the products from DCL1 processing during miRNA biogenesis in at least one of the libraries or the mature sequence could be identified from more than half of our libraries.

### Target gene prediction for miRNAs and KEGG pathway analysis

To determine the potential targets of known and novel miRNAs expressed in rice grain filling, Oryza Sativa MSU Rice Genome Annotation release 6.1 was used. The rules used for target prediction were based on those suggested by Allen et al [31] and Schwab et al [32]:1) No more than four mismatches between miRNA and potential target (G-U bases count as 0.5 mismatches); 2) No more than two adjacent mismatches in the miRNA/target duplex; 3) No adjacent mismatches in positions 2–12 of the miRNA/target duplex (5′ of miRNA); 4) No mismatches in positions 10–11 of miRNA/target duplex; 5) No more than 2.5 mismatches in positions 1–12 of the miRNA/target duplex (5′ of miRNA); 6) Minimum free energy (MFE) of the miRNA/target duplex should be ≥75% of the MFE of the miRNA bound to its perfect match location. Then, online genomic analysis tools of the KEGG pathway database (http://www.genome.jp/kegg/) were used for pathway enrichment analysis.

### DGE library construction, sequencing and digital tag profiling

Sequence tag preparation was performed with the Illumina Digital Gene Expression Tag Profiling Kit (Illumina, Inc.) according to the manufacturer's protocol. In brief, total RNA (6 µg) isolated from rice grains at 10DAF, 15DAF, 21DAF, 27DAF, and 35DAF were used to purify mRNA by OligodT magnetic beads adsorption, respectively, then the first and second-strand cDNA were synthesized, and the 5′ ends of tags were generated by endonuclease Nla III, which recognizes and cuts off the CATG sites. The fragments apart from the 3′ cDNA fragments attached to OligodT beads were washed away. The Illumina adaptor 1 was ligated to the sticky 5′ end of the digested bead-bound cDNA fragments, which containing a recognition site for the endonuclease Mme I which had the ability to cut 17 bp downstream from the recognition site (CATG). After removing 3′ fragments with magnetic beads precipitation, Illumina adaptor 2 was ligated to the 3′ ends of tags, acquiring tags with different adaptors of both ends to form a tag library. After 15 cycles of linear PCR amplification, 105 bp fragments were purified by 6% (w/v) TBE PAGE Gel electrophoresis. Followed by denaturation, the single-chain molecules were fixed onto the Illumina Sequencing Chip (flowcell). Each molecule grows into a single-molecule cluster sequencing template through situ amplification. Then four types of nucleotides, which are labeled by four colors, were added and sequencing with the method of sequencing by synthesis was performed.

After sequencing, the raw sequences were transformed into 17 bp clean tags, and tag counting was carried out using the Illumina Pipeline. First, 3′ adaptor sequence and low quality tags, including short tags (<21 nt), long tags (>21 nt), empty reads, and the tags which were sequenced only once were removed. Then the remaining clean tags were mapped to the rice genome by SOAP [28]. The gene expression levels were represented by a summation of all tags while multiple tag types aligned to different positions of the same gene. Then the gene expression data were normalized to transcripts per million (TPM) clean tags.

### Quantitative real-time RT-PCR (Q-PCR)

Q-PCR experiments were preformed as we used previously [30]. In brief, total RNA (1 µg) was reverse-transcribed using a reverse transcriptase enzyme (Promega) and the test miRNA-specific stem-loop reverse transcription primer. In the Q-PCR, a 5 µl aliquot of 1∶20 diluted cDNA was used as the template in a 20 µl PCR reaction system. They were carried out using SYBR green reaction mix (SYBR® Green QRT-PCR Master Mix; Toyobo) after a pre-incubation at 95°C for5 min, followed by 40 cycles of denaturation at 95°C for15 s, annealing at 60°C for 15 s, and extension at 72°C for 32 s, in a BioRad iQ5 sequence detection system (BioRad, USA). The actin gene was used as the house-keeping gene. The relative fold expression changes of target genes were calculated using the 2 delta-delta Ct method. All gene specific primers used in the experiments are listed in **Table S1**.

### RNA ligase-mediated 5′ RACE

To map the cleavage sites of target transcripts, we used RNA ligation-mediated (RLM) rapid amplification of 5′ complementary DNA ends (5′ RACE) using a GeneRacer kit (Invitrogen, Carlsbad, CA, USA). In brief, total RNAs (4 µg) from equal mixtures of rice grains (10DAF, 15DAF, 21DAF, 27DAF, and 35DAF) were ligated to a 5′ RACE RNA adapter without calf intestine alkaline phosphatase treatment. cDNAs were transcribed with reverse transcriptase by the GeneRacer OligodT primer. GeneRacer™ 5′ Primer (5′-CGACTGGAGCACGAGGACACTGA-3′), GeneRacer™ 5′ Nested Primer (5′-GGACACTGACATGGACTGAAGGAGTA-3′) and two gene-specific reverse primers were used for each RACE (**Table S1**). In each case, a unique gene-specific DNA fragment was amplified. These products were gel purified, cloned (pGEM-T Easy, Promega), and 7 independent clones from each reaction were sequenced finally.

## Results

### Global analysis of small RNAs in rice grain filling

To investigate the dynamic variation of miRNAs during rice grain filling, Solexa high-throughput sequencing technology was employed to sequence the small RNAs from the immature grains at 10DAF, 15DAF, 21DAF, 27DAF, 35DAF. After removing the low quality sequences and adapter sequences, a total of 10,658,388 to 14,248,902 reads, representing 3,518,252 to 4,770,477 unique sequences were obtained in each library (**Table 1**). Furthermore, more than 79.68% of the unique small RNAs, standing for over 82.46% of the small RNAs mapped perfectly to the genome (**Table 1**). To find the known miRNAs and their expression at different periods of rice grain filling, the clean reads of each datasets were mapped to the precursor sequences of rice miRNA database available in miRBase (release 17). Finally, we obtained 1,073,728 to 1,146,855 tags, representing 5,701 to 5,941 unique reads mapped perfectly to the miRNA precursor in the five grain-filling stages. In addition, small RNAs from Solexa deep sequencing also contained other kinds of RNAs, including rRNA, tRNA, snRNA and snoRNA with different abundances (**Table 1**).

**Table 1:** Summary of small RNA classes during rice grain filling.

Category	10DAF		15DAF		21DAF		27DAF		35DAF	
	Unique	Total	Unique	Total	Unique	Total	Unique	Total	Unique	Total
**Total**	4,478,689	14,248,902	4,770,477	13,297,350	3,930,540	11,378,685	3,884,446	11,647,614	3,518,252	10,658,388
**exon_antisense**	133,165	746,308	133,212	804,510	118,578	739,153	123,673	894,862	114,010	871,834
**exon_sense**	219,967	757,184	228,286	616,801	220,097	503,337	218,837	499,487	190,991	453,826
**intron_antisense**	130,900	452,915	140,388	346,714	115,639	218,944	116,330	198,276	102,339	174,627
**intron_sense**	138,967	500,657	151,203	435,494	124,176	294,757	127,163	282,784	112,208	261,087
**miRNA**	5,693	1,073,707	5,932	1,146,820	4,768	1,114,668	4,985	1,141,378	4,930	1,145,735
**rRNA**	78,926	1,143,956	74,912	1,104,228	88,092	1,653,911	95,275	1,798,049	88,828	1,416,786
**repeat**	1,335,756	3,100,009	1,506,174	3,275,580	1,247,244	2,539,845	1,258,742	2,432,808	1,138,203	2,248,862
**snRNA**	1,623	3,734	1,246	2,260	1,258	2,695	1,319	2,734	1,191	2,368
**snoRNA**	2,729	5,431	2,320	4,368	1,926	3,423	2,045	3,535	1,798	3,127
**tRNA**	8,815	270,468	10,435	391,405	10,829	573,145	12,300	664,809	12,391	474,220
**No_annotation**	2,422,148	6,194,533	2,516,369	5,169,170	1,997,933	3,734,807	1,923,777	3,728,892	1,751,363	3,605,916

**Figure 1:**
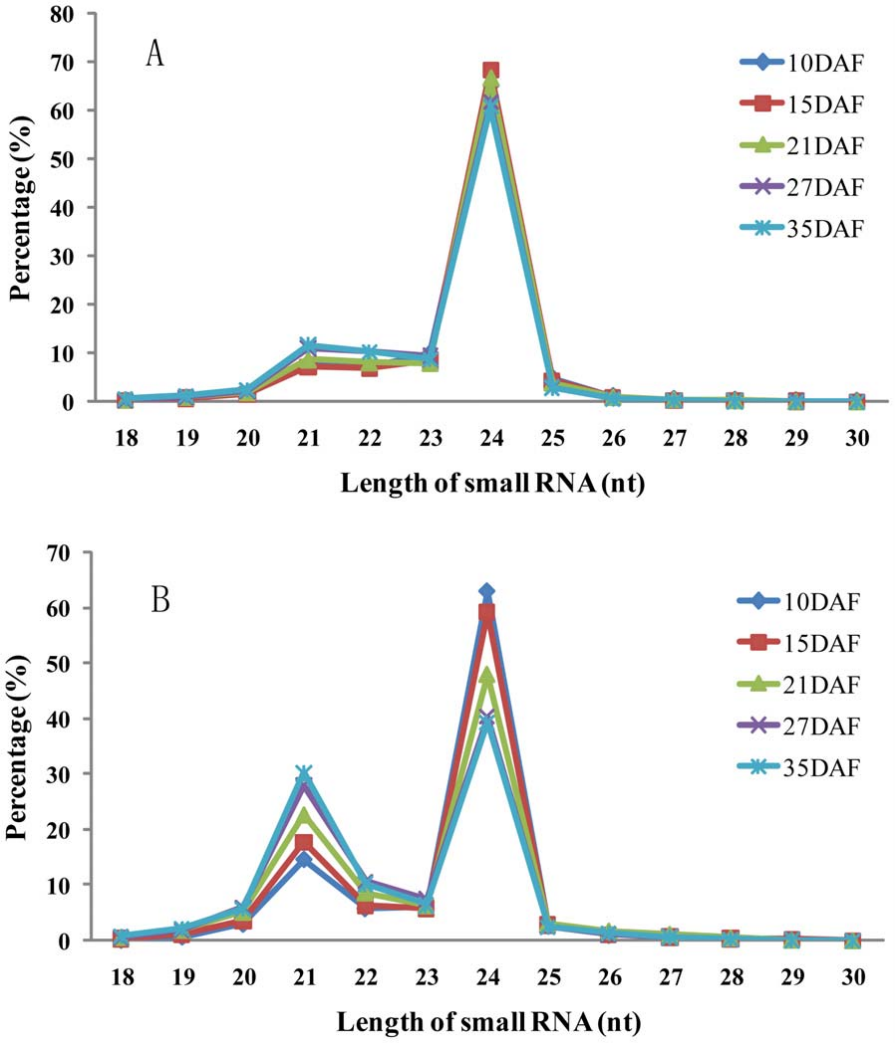
Size distributions of the unique (A) and total (B) small RNA sequences during rice grain filling.

In plants, the majority of small RNAs are 21 to 24 nt in length, most miRNAs are 20 to 22 nt; siRNAs are mostly 21 and 24 nt [33], whereas 23 nt small RNAs may present as a new class of functional small RNAs [23]. In this study, the numbers of unique and total small RNA sequences ranging from 18 to 30 nt in length was examined. When the unique small RNA sequences were counted, 24 nt small RNAs were dominant in all libraries (**Fig. 1A**). In terms of total small RNA sequences from the five libraries made from different stages during rice grain filling, there are two peaks at 24 and 21 nt, and more than half of them were 24 nt, accounting for 50.85%, while 21 nt small RNAs shared 22.07%. Moreover, the percentage of 24 nt small RNAs gradually decreased with rice grain filling, while 21 nt small RNAs showed a reverse trend (**Fig. 1B**).

### Newly identified miRNAs involve in rice grain filling

In general, the presence of miRNA*s are indicative of *bona fide* miRNA genes in plants [34]. Therefore, novel miRNAs identified from the miRNA candidates produced from MIREAP were first considered based on the presence of miRNA*s in at least one of our libraries, and 22 novel miRNAs were identified by this standard. On the other hand, 23 novel miRNAs were founded based on a different method proposed by Wei et al [35] as we described in materials and methods. In total, 45 novel miRNAs were identified in this study (**Table 2** **,Fig. S1**). Among the novel miRNAs, some of them were expressed only in specific periods of grain filling. For instance, miRn1-n17 were highly accumulated in one or two phases, but not expressed in other stages during rice grain filling. In addition, miRn45-5p abundantly detected in all the five libraries may have physiological effects on rice grain filling (**Table 2**).

**Table 2:** Sequences and abundance of novel identified miRNAs during rice grain filling.

miRNA ID	Squence	Length	Abundance (TPM)					Evidence#
			10DAF	15DAF	21DAF	27DAF	35DAF	
miRn1-5p	CGGCGATGTGGGGGAGGCGCG	21	5.47	0	0	0	0	*
miRn2-5p	AACACACCGGATTCGAATCTTTT	23	0.49	0	0	0	0	*
miRn3-5p	TCGACCACGTCGAAGAGGCTCA	22	0	0.45	0	0	0	*
miRn4-5p	TCAACTTCGTTTCGTGAGGCA	21	0	0	0.79	0	0	*
miRn5-5p	CGGTGACAGAAGAGAGTGAGCAC	23	0	0	0	0.43	0.47	*
miRn6-5p	TTAGTCTACGTTTCATCTCTC	21	0	0	0	1.2	0	*
miRn7-3p	TCTGCTAATGGTGGTCAGGAC	21	0	0	0	1.03	1.59	*
miRn8-3p	TAGGATGAGAGTTGCATGGTT	21	0	0	4.22	0	0	*
miRn9-5p	AAGGGGCGCTTACTGAGAGTTCT	23	0	0.38	0	0	0	*
miRn10-5p	TCTTATTGGGTCGTGCCTAGGC	22	1.4	0.83	0	0	0	*
miRn11-5p	TGTGTAGCCACATTGTAAGGG	21	0.35	0	0	0	1.13	*
miRn12-5p	TGGCCTGTAGTTAGTAGAGGG	21	0	0	0.53	0	0	*
miRn13-3p	TCAGCACGGATACATTATTTT	21	0.42	0	0	0	0	*
miRn14-3p	TGGGAATATTCTTTATCTGTT	21	0	0.83	0	0	0	*
miRn15-5p	TAAATGGAGAGAACGAAAGAG	21	0	0	4.39	0	0	*
miRn16-3p	TCGGACTCTCGGCGGCGCTCG	21	0	0	0	0	0.84	*
miRn17-5p	TCTTTCACATGGTATTAGAGCTG	23	0	0.98	1.05	0	0	*
miRn18-3p	TAGATATATATCTAGGATTGGAT	23	0.77	1.43	0	0.94	1.31	*, T
miRn19-3p	TAACAAAGGACAACAGACTGA	21	1.89	0.98	0.88	0.86	1.59	*, T
miRn20-3p	CTTTGAGTAGGGTCTAAACAGAG	23	7.16	12.86	12.83	7.38	9.38	*, T
miRn21-5p	CAGGCAGAGCATGAAGAGCAT	21	0	1.13	1.05	1.29	0	*, T
miRn22-3p	TACATTTGGAACCAGAGGGAC	21	3.65	4.51	14.24	19.49	17.17	*, T
miRn23-5p	TGAGGACAAGAGCTGATTCGG	21	6.46	3.84	9.84	3.18	1.03	T
miRn24-3p	TTTGAACTTGATATTTGGTGG	21	1.75	1.96	1.32	1.03	1.22	T
miRn25-3p	TACGAGAGATGGGAAAAGACAAC	23	1.12	2.41	1.32	1.29	0.66	T
miRn26-5p	TTTCTTGACTCGGGATGACTAG	22	1.33	1.2	0	0.69	0.75	T
miRn27-5p	TCGGGATATGTGGTATTGCGGTT	23	0.84	0	1.05	1.29	0	T
miRn28-5p	CACGGAATGGAAGAGCGAGAC	21	0.7	1.13	0	0	4.03	T
miRn29-5p	ACCCCCCTTTGCCGGCGCGCGC	22	0.49	0.75	0	0.6	0.47	T
miRn30-5p	TGAAAAGTTGGGAATTTGGAG	21	0.77	0	0.62	0.69	0.47	T
miRn31-5p	TGGGTTTGGGTGGTGGTGGTGCA	23	2.67	3.01	0.97	0	0.66	T
miRn32-5p	TAGATGGCTGATCTGGTGTGG	21	1.68	0	2.72	2.66	4.03	T
miRn33-3p	TGAGATTGGTTTATTTTGGGA	21	1.05	1.88	1.49	1.72	0	T
miRn34-5p	TTCGTAAGTGGAACCGCACGG	21	0.42	0	0	3.26	0.84	T
miRn35-3p	TCAGGAGCAGAAGATGAGGGAG	22	2.39	5.11	4.57	5.41	4.5	T
miRn36-5p	TCTCACTTTGGACTAGGTATT	21	4.35	8.12	10.81	11.59	11.63	T
miRn37-3p	TTGGTAAGGCGAAAATTGGCAT	22	1.82	1.13	1.49	1.89	1.78	T
miRn38-3p	TTGGGGAACGCGCCGATCGTC	21	0.63	0	3.16	1.97	2.06	T
miRn39-5p	TGCTCCGGATATTATGGCATG	21	4.63	5.11	2.81	3.61	6.29	T
miRn40-3p	TCTGAGACAGCGTAGACAGTT	21	1.75	1.73	1.58	0	0	T
miRn41-3p	TGCGAAGTAGAGATGCCGACT	21	0	1.73	1.14	0.94	0	T
miRn42-5p	ATCTCGATGGTGATTGTTGCT	21	0	1.43	0	0.6	0.47	T
miRn43-3p	TTTTGGGAGGATGGCAAATAG	21	0	0.83	0.79	0.77	0	T
miRn44-3p	TCGATGCAGTCCTCGATGTCG	21	0	0.53	0	0.52	1.31	T
miRn45-5p	GCTGGAGTAGCTCAGATGGT	20	93.06	175.82	363.22	317.49	596.71	T

# Evidence of identified miRNA, ‘*’ indicates the detection of the corresponding miRNA. ‘T’ indicates that the miRNA were founded at least in three of the five libraries.

### The finding of miRNA*s with an abundance higher than their corresponding miRNAs

**Figure 2:**
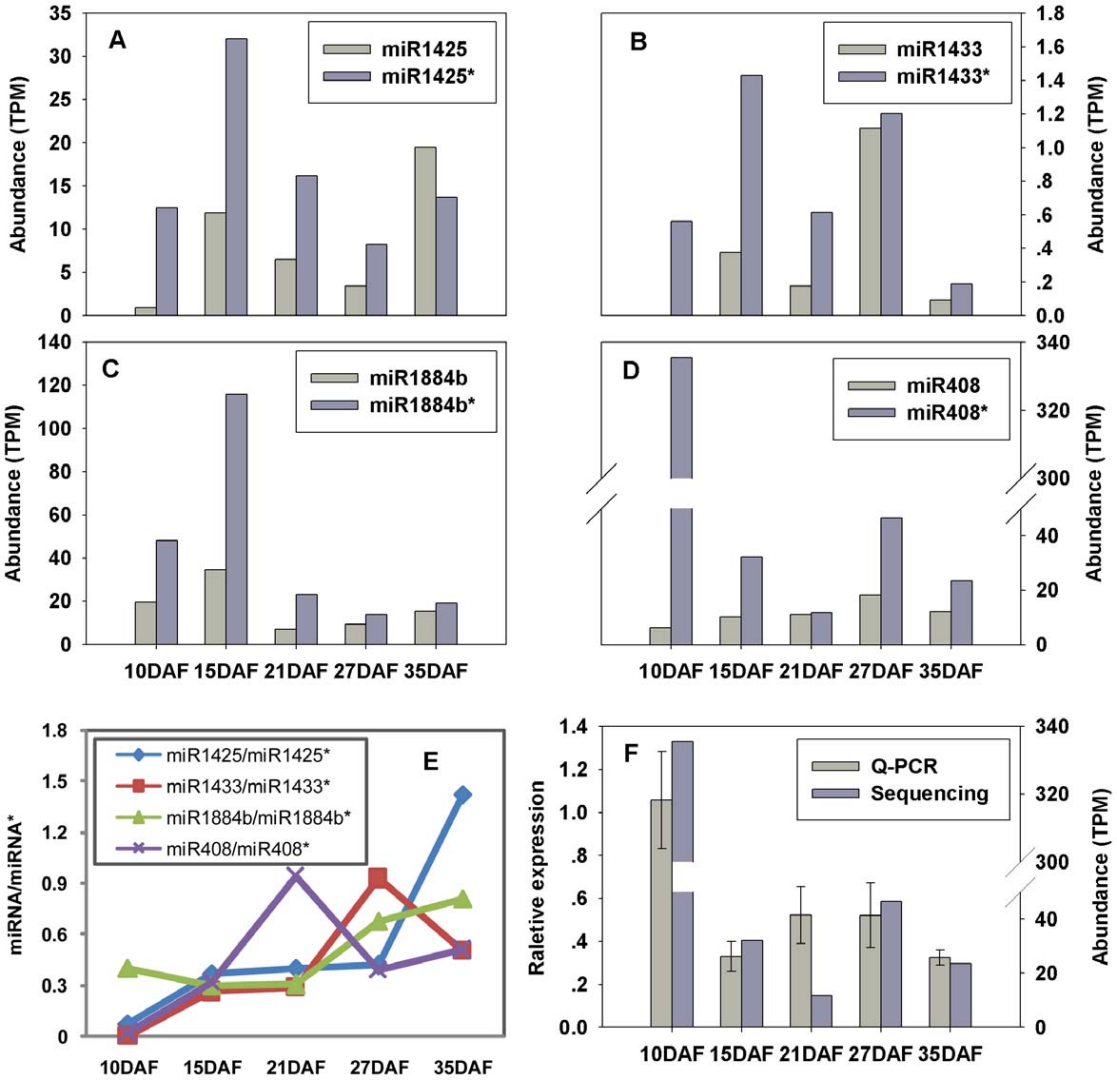
Expression of several miRNAs and their corresponding miRNA*s. **A–D:** Normalized sequence reads of miR1425, miR1433, miR1884b, miR408, and their corresponding miRNA*s during rice grain filling. **E**: The ratio of miRNAs and their corresponding miRNA*s. **F**: validation of the abundance of miR408* by Q-PCR during rice grain filling. DAF represents days after flowering.

Under normal conditions, miRNA/miRNA* duplexes are unwound after being exported out of the nucleus. MiRNA strands are loaded into the RNA-induced silencing complexes (RISCs) and the miRNA* strands are degraded [12], [36]. Therefore, miRNA levels are usually higher than their corresponding miRNA* levels. However, in our datasets, four miRNA*s (miR1425*, miR1433*, miR1884b*, and miR408*) were more abundant than their corresponding miRNAs, with the most abundant miR408* at more than 50 fold over miR408 at 10DAF (**Fig. 2** **, Fig. S2**), which is consistent with our previous results [30]. But these four miRNA*s were not always higher than their corresponding miRNAs. For example, miR1425 is higher than miR1425* at later grain filling stage of rice grain filling (35DAF, **Fig. 2**). A recently developed degradome sequencing approach has been successfully used for validating the targets of rice miRNAs [37]–[39]. To analyze the functions of these highly expressed miRNA*s, starBase [40] was employed to find the targets of these miRNA*s in previous degradome sequencing data. Excitingly, four targets of miR1425*, miR1433*, and miR1884b* were identified and validated by the presence of a great many degradation products in the degradome sequencing dataset (**Table S2**). These results are consistent with the previously researches [41], [42] and indicate that these miRNA*s may play important physiological roles in rice grain filling.

### Dynamics analysis of known miRNAs during rice grain filling

**Figure 3:**
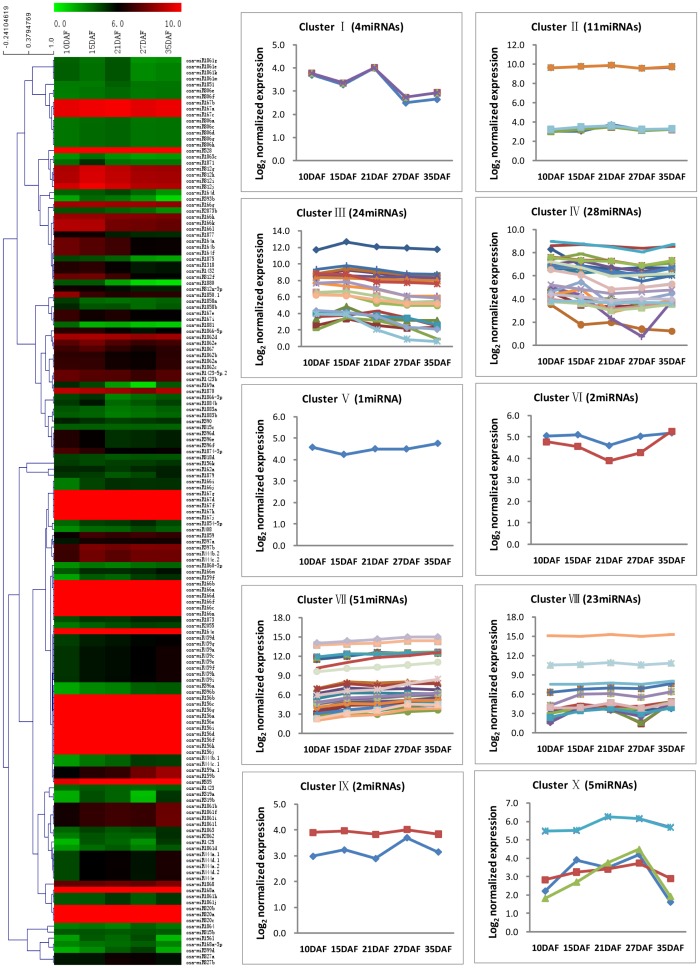
Hierarchical clustering and expression patterns of known miRNAs during rice grain filling. miRNAs that are expressed higher than 10 TPM in one of our datasets were listed, depicted in a heat map representation, and performed on the log_2_-normalized expression. Red represents high expression and green represents low expression. The line charts showed the expression patterns of every cluster in the heat map on the left. DAF represents days after flowering.

**Figure 4:**
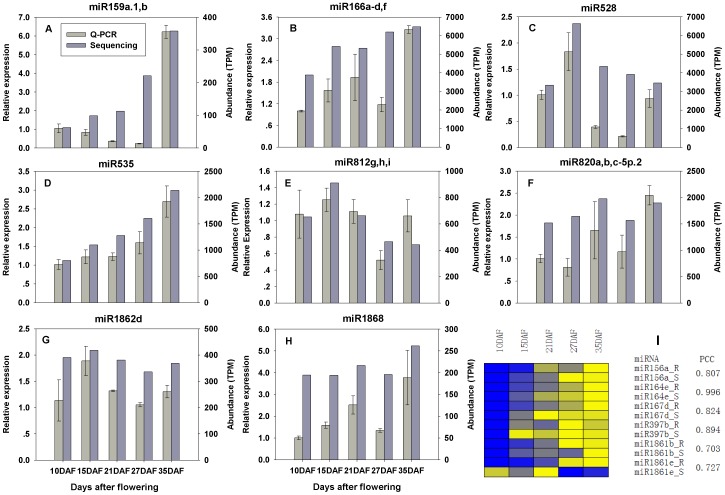
Expression profiling analysis and validation of the abundance of miRNAs by Q-PCR during rice grain filling. **A–H**: The expression levels of each miRNA were normalized by comparison with the expression of miRNA in immature grains at 10DAF, which was set as 1.0. The experiments were repeated with three biological replicates and error bars represent the standard error. **I**: Correlation of the abundance between Q-PCR (we assayed previously [30]) and Solexa sequencing was presented by Pearson's correlation coefficient (PCC). Yellow represents high expression and blue represents low expression. R denotes expression detected by Q-PCR and S represents expression detected by Solexa sequencing.DAF represents days after flowering.

In total, 445 known miRNAs were found in the five libraries (**Table S3**). Then, the miRNAs whose expression were higher than 10 TPM in one of our datasets were filtered, and a total of 151 known miRNAs remained for further analysis to minimize noise and improve accuracy (**Table S4**). After hierarchical clustering, these miRNAs were classified into ten groups, and a high proportion of known miRNAs were constitutively expressed during rice grain filling (**Fig. 3** **; Table S5**). The major representative clusters of this category were VII, IV, III and VIII, which contain 51, 28, 23 and 23 miRNAs, respectively. The miRNAs in cluster VII showed gradually enhancing expression during rice grain filling, but they were negatively correlated with the grain filling rate [PCC (Pearson's correlation coefficient) ≤−0.554, **Fig. 3** **; Fig. S3; Table S5**]. While miRNAs in cluster IV first slightly decreased and then increased at later grain filling stage (35DAF), they were positively correlated with the grain filling rate (PCC≥0.532, **Fig. 3** **; Fig. S3; Table S5**). In cluster III, the expression pattern of these miRNAs showed a gradual increase at early grain filling stage (10–15DAF) and slightly decreased thereafter, showing positive correlation with the grain filling rate (**Fig. 3** **; Fig. S3; Table S5**). In addition, the miRNAs in cluster VIII showed a gradual increase until the middle grain filling stage (25DAF) then decreased, and finally increased, showing negatively correlation with the grain filling rate (**Fig. 3** **; Fig. S3; Table S5**). To validate the expression profiles determined by using Solexa sequencing, we performed stem-loop real time quantification RT-PCR (six of them were assayed previously [30]). While the expression patterns of almost all of the tested miRNAs were consistent with those detected by Solexa sequencing during rice grain filling, discrepancies were also observed in the expressions of a few other miRNAs. Such discrepancies may be due to variations in sampling time and methods to be used (**Fig. 4**).

### Target prediction and validation of known and novel miRNAs

**Figure 5:**
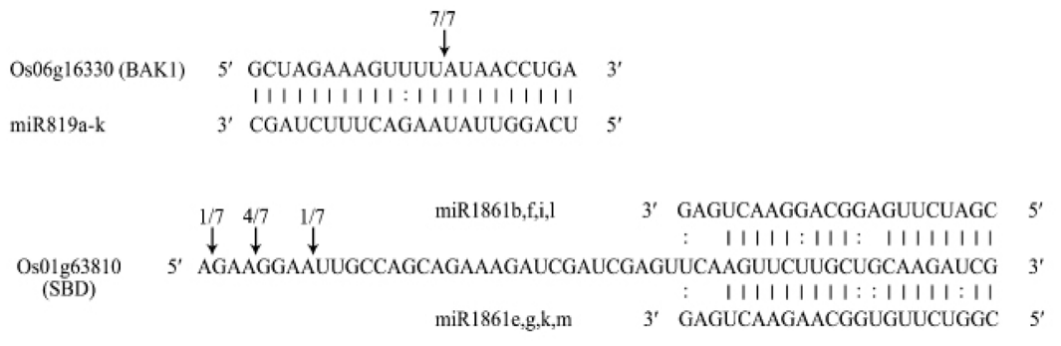
5′-RACE verification of predicted miRNA targets. Watson-Crick pairing (vertical dashes) and G:U wobble pairing(colons) are indicated. Arrows indicate the 5′termini of mRNA fragments isolated from the mixture of immature rice grains, and the frequency of clones was shown on the top of the arrow. BAK1: BRASSINOSTEROID INSENSITIVE 1-associated receptor kinase 1 precursor; SBD: starch-binding domain containing protein.

In target gene prediction, 133 highly expressed known miRNAs (>10 TPM) were predicted to target 2,942 genes (**Table S6**). KEGG analysis showed these target genes participated in many biological pathways, such as starch and sucrose metabolism, fructose and mannose metabolism, brassinosteroid biosynthesis, zeatin biosynthesis and so on, and played crucial roles in rice grain filling (**Table S7**). To validate the potential targets of miRNAs that might play crucial roles in rice grain filling, two predicted target genes, as an example, were selected and assayed by using rapid amplification of 5′ cDNA ends (5′ RACE). Os06g16330 encoding a BRASSINOSTEROID INSENSITIVE 1-associated receptor kinase 1 precursor (BAK1) was confirmed as the real target of miR819a-k, which is involved in brassinosteroid homeostasis in plants, and seven cleavage events were detected in the miRNA target site (**Fig. 5**). Os01g63810 is the predicted target of miR1861, encoding a starch-binding domain-containing protein. However, the main cleavage site detected was 29 nt upstream of the predicted miR1861 target site, which was probably caused by an unknown small RNA cleavage mechanism (**Fig. 5**). In addition, 422 genes were predicted to be the targets of 31 novel miRNAs (**Table S8**). Furthermore, six targets for five novel miRNAs were validated by degradome data in starBase (**Table S9**) [40]. However, the cleavage products of these targets showed at a very low frequency in the degradome data. This might be due to the fact that these novel miRNAs were expressed in a tissue-specific and/or developmental stage dependent manner, and that the degradome data was from collective samples of different organs or development stages.

### Correlation analysis of expressions between miRNAs and their corresponding targets

**Figure 6:**
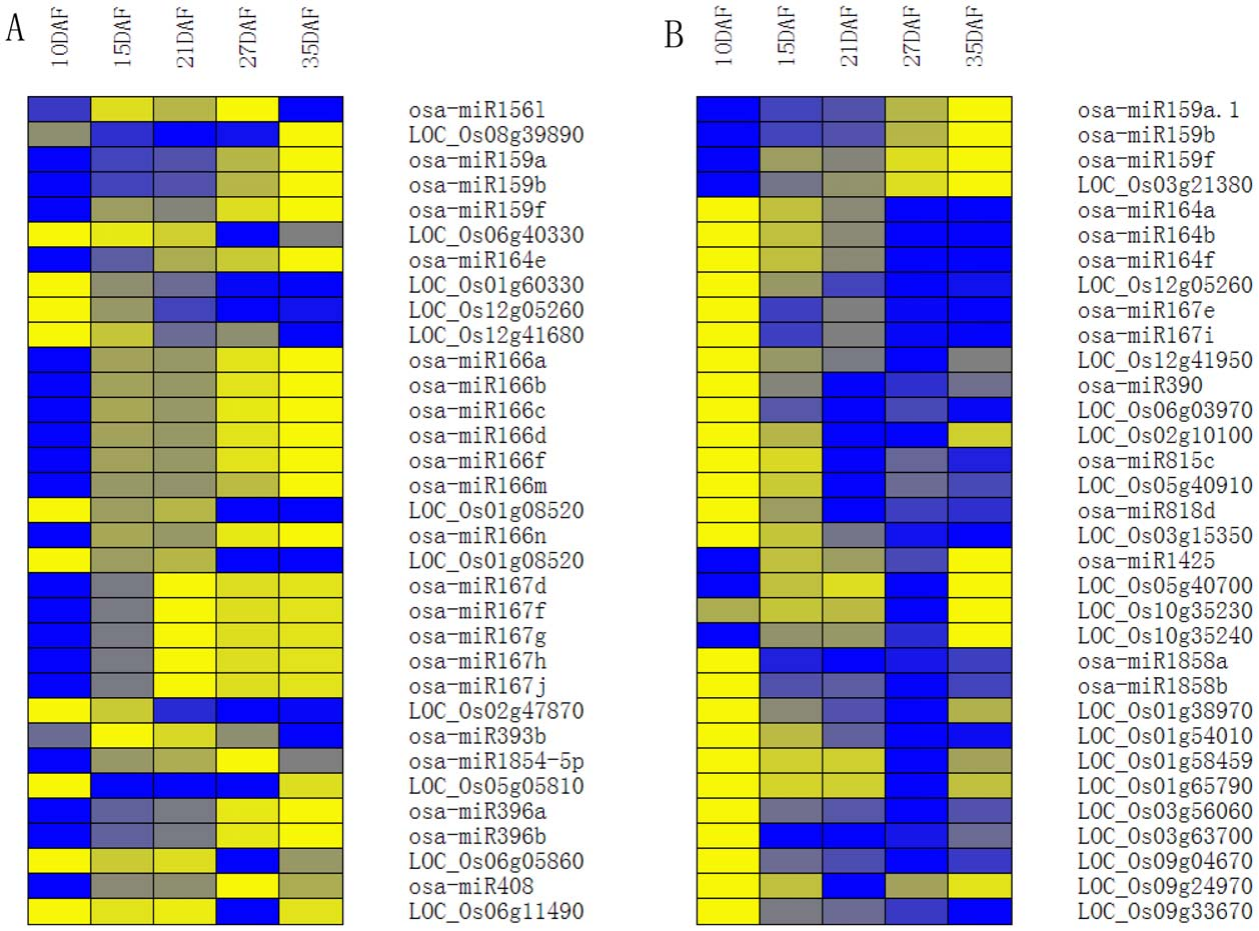
Expression correlations of miRNAs and their correspondingtargets during rice grain filling. (A) The expression of miRNAs showed negative correlations with their corresponding targets during rice grain filling. (B) The expression of miRNAs showed positive correlations with their corresponding targets during rice grain filling. Yellow represents high expression and blue represents low expression. The heap map was performed on the log2-normalized expression. DAF represents days after flowering.

In general, the expression of most miRNAs were negatively correlated with their targets [25]. To further study the relationship between miRNAs and their targets, digital gene expression profiling (DGE) was performed to assay the expression of target genes during rice grain filling. In total, 157 out of 270 target genes identified via degradome sequencing in starBase showed a negative correlation with their corresponding miRNAs (**Fig. 6A** **; Table S10**), which is consistent with negative correlation of most miRNAs with their corresponding targets in rice reported by Xue et al [25], indicating that the majority of miRNAs guided the degradation of their target gene transcripts. However, some miRNAs and their target genes showed differential or a positive correlation in expression patterns during rice grain filling. For instance, miR159a.1,b,f, miR164a,b,f, miR167e,i, miR390,miR815c, miR818d, miR1425, miR1858a,b were positively correlated with their targets (**Fig. 6B** **; Table S10**), which may be due to a similar feedback regulation between co-expressed MIR164A and CUC2 genes (miR164 target) as reported in Arabidopsis [43]. It is also possible that these miRNAs might function as translational repressors [36], as the case of miR167 reported in Arabidopsis [44].

## Discussion

MiRNA as a new class of regulatory factors has attracted much attention in recent years. Despite 547 miRNAs and one miRNA* being reported in Oryza sativa according to the miRBase (release 17), regulatory roles of miRNAs in rice grain filling is poorly understood. In this work, high-throughput sequencing technology was employed to investigate the known and novel miRNAs and their expression pattern analysis during rice grain filling. In parallel, the expression patterns of higher expressed miRNA target genes identified through degradome sequencing in starBase were also studied by DGE during rice grain filling, providing the first miRNA dynamics for rice grain filling.

### Expression patterns of miRNAs during rice grain filling

It is well known that miRNAs are expressed in a developmental stage-dependent and/or tissue-specific manner [12], [35], [45]. Through deep sequencing, we found that the expression of almost all miRNAs varied with the development of grain filling. But, the majority of these miRNAs increased gradually with the progress of grain filling process (cluster VII). These findings are in agreement with the majority of miRNAs that were expressed higher in 6 to 10 DAF grains than in 1 to 5 DAF grains and about half of the detected miRNAs were up-regulated with the grain filling process of indica rice [20], [24]. In addition, 45 novel miRNAs identified in this work displayed expression in a developmental stage dependent manner. Furthermore, the expression of the miRNAs incluster III and IV were positively correlated with the grain filling rate, whereas miRNAs in cluster VII and VIII were negatively related to the grain filling rate (**Table S5**). As a result, we conclude that the expression dynamics of these miRNAs found in the grain filling might play key roles in regulating many metabolic processes involved in the rice grain filling through cleaving or translationally repressing their corresponding target gene transcripts in a grain filling stage-dependent manner.

### miRNA* may involve in regulating rice grain filling

It is believed that miRNA*s were gradually degraded when mature miRNAs guided the silencing of their corresponding target mRNAs with the help of RNA-induced silencing complex (RISC) [36]. Nevertheless, four miRNA*s (miR1425*, miR1433*, miR1884b*, and miR408*) were more abundant than their corresponding miRNAs were found (except for miR1425 at 35DAF), and the ratio between miRNA and miRNA* varied during rice grain filling as well (**Fig. 2**). In recent years, cleavage and translational repression of miRNA and miRNA* have been already observed in plants through two distinct argonaute proteins (AGOs): miRNA through AGO1 and miRNA* through AGO2 [46], [47]. One example is miR393b*, which regulates plant immunity by translational inhibition its targeting gene MEMB12 involved in plant defense responses through AGO2 in Arabidopsis [47]. miR169d*,l*,m*,e.2*, more abundant than miR169, mediate the cleavage of the arbuscule-specific protein MtBcp1, and miR5024* targets a GRAS transcription factor specifically expressed in mycorrhizal roots of Medicago truncatula [41]. In addition to the previous reported miRNA*s, four target genes regulated by miR1425*, miR1433* and miR1884b* were validated by previous degradome sequencing (**Table S2**). For example, miR1433* targets two nuclear transcription factor Y subunits shown to control a variety of important agronomical traits, including seed development, flowering time, and drought tolerance [48]. Moreover, miRNA*-mediated self-regulation of their host precursors has been discovered in Arabidopsis and rice, such as ath-MIR161 and osa-MIR445d [49]. Furthermore, miRNA/miRNA* ratios different during rice grain filling was founded, which also exists in mammals [50], where miR-223 and miR-223* targeted the same mRNA and higher miR-223* levels were associated with increased overall survival in patients with acute myeloid leukemia [51]. Hence, higher levels of miRNA* may be involved in rice grain filling through directing cleavage or translational repression of their target mRNAs, self-regulation of their host precursors, or functioning dependent on the ratio between the mature and star strands.

### MiRNA regulates plant hormone homeostasis during rice grain filling

**Figure 7:**
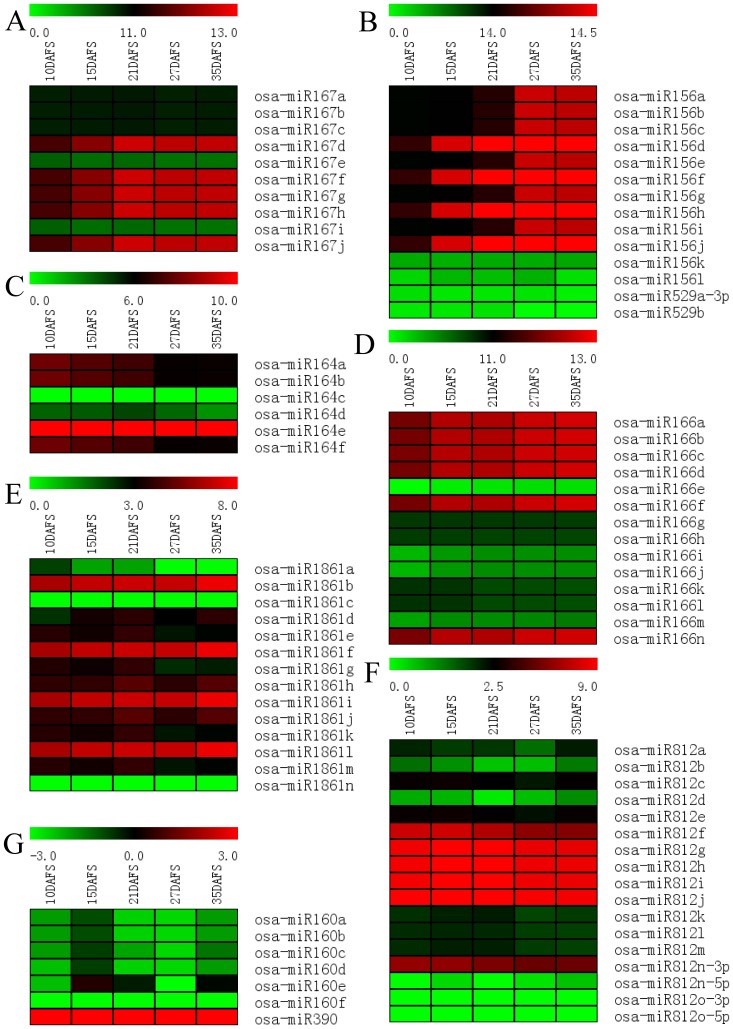
Expression analysis of miRNAs involve in rice grain-filling. Red represents high expression and green represents low expression. The heap map was performed on the log2-normalized expression. DAF represents days after flowering.

**Figure 8:**
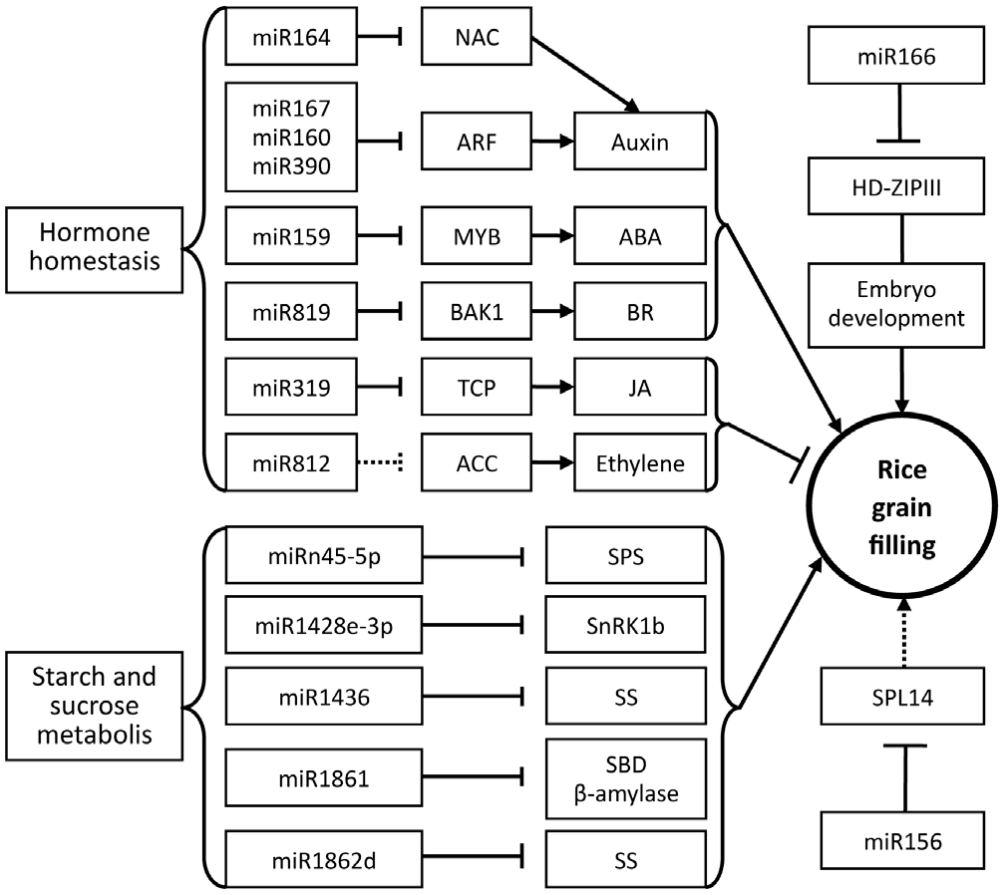
A possible functional network of miRNAs involved in rice grain-filling. The arrows indicate positive regulation, the nail shapes represent negative regulation, and straight lines indicate regulation. ARF: auxin response factor; MYB: MYB family transcription factor; TCP: TCP family transcription factor; ACC: 1-aminocyclopropane-1-carboxylate oxidase protein; BAK1: BRASSINOSTEROID INSENSITIVE 1-associated receptor kinase 1 precursor; JA: jasmonic acid; BR: brassinosteroid; SPS: sucrose-phosphate synthase; SBD: starch-binding domain containing protein; SnRK1: sucrose non-fermenting-1-related kinase 1; SS: starch synthase.

Several plant hormones, including auxin, abscisic acid, cytokinin, gibberellin, ethylene, brassinosteroids, and jasmonate, take part in many biological processes throughout plant growth and development [52]. And without exception, these hormones play key roles in regulating rice grain filling [7]–[9], [53]. Accumulating evidence indicates that miRNAs play vital roles in plant phytohormone homeostasis [15], [16], [54]–[56], suggesting a miRNA-phytohormone interaction might have roles in rice grain filling post pollination/fertilization. For example, OsGH3-2 is a rice IAA-conjugating enzyme, and miR167 down regulated the expression of OsGH3-2 by cleavage of ARF8 mRNA in the presence of auxin. This indicates that the auxin-miR167-ARF8-OsGH3-2 pathway is indispensable in regulating the appropriate cellular free auxin level in plants [56]. In our dataset, miR167 was strongly expressed in the developing stage of rice grains, as well as gradually increased during rice grain filling periods (**Fig. 7A**). Similar trends were observed in miR156, miR164, miR166, and miR1861 (**Fig. 7B–E**), but were not apparent for miR812 (**Fig. 7F**). Furthermore, during rice grain filling, the dynamic changes of endogenesis IAA shows a negative correlation with the expression of miR167, but positive correlation with the grain filling rate [9]. Thus, the miR167-ARF8-OsGH3.2-IAA pathway, in conjunction with other microRNA-mediated auxin signals, such as miR164 [57], miR160 [58], [59], and miR390 [55] results in a suitable IAA level in developing rice grains for regulating the progress of rice grain filling (**Fig. 8**).

MiR159, miR319, miR812, and miR819 target gene transcripts coding MYB family transcription factor (MYB), TCP family transcription factor (TCP), 1-aminocyclopropane-1-carboxylate oxidase protein (ACC), and BRASSINOSTEROID INSENSITIVE 1-associated receptor kinase 1 precursor (BAK1), which are involved in the regulation of ABA, jasmonic acid, ethylene, and brassinosteroid homeostasis, respectively [21], [54], [60]. During rice grain filling, the content of ABA was positively correlated with cell division and grain filling rate, while ethylene showed a reversed trend [7], [9]. Application of cobalt ion (inhibitor of ethylene synthesis) or ABA at an early grain filling stage significantly increased endosperm cell division rate and cell number, grain filling rate, and grain weight of inferior spikelets, while application of ethephon or fluridone (an inhibitor of carotenoid synthesis) had the opposite effect [7]. Furthermore, jasmonic acid and brassinosteroid were also reported to affect grain filling in rice (**Fig. 8**) [8], [61]. In addition, miRNAs involved in zeatin biosynthesis have been identified by KEGG pathway analysis in this study and may play important roles in determining the cellular free cytokinin level (**Table S7**). Rates of endosperm cell division and grain filling were reported to have a positive correlation with zeatin (Z+ZR) contents in rice grain development; application of kinetin to spikelets increased the total cell numbers per endosperm, grain filling rate, and grain weight of inferior spikelets (**Fig. 8**) [9], [62].

### MiRNAs involved in regulating genes for starch synthesis during rice grain filling

Rice grain filling is a process of starch biosynthesis in the growing endosperm. It has been reported that a series of enzymes, such as SUS, AGPase, SS, and SBE, play key roles in the pathway of starch and sucrose metabolism and are significantly correlated with rice grain filling rate [6], [63]. Several miRNAs were found to have differential expression that may have an impact on their target genes participating in starch and sucrose metabolism in this study. For example, A novel miRNA, miRn45-5p, was validated to target a putative sucrose-phosphate synthase (SPS), a key enzyme in sucrose synthesis by degradome data in starBase (**Table S9**) [64]. Os01g63810, encoding a starch-binding domain containing protein (SBD), is targeted by a miR1861 family that was strongly accumulated during rice grain filling (**Fig. 7E**), while transcript-encoding β-amylase (Os10g32810), targeted by miR1861, has been validated by degradome data [65]. Two other miRNAs, namely miR1436 and miR1862d, both cleaving mRNAs encoding starch synthase (SS), have been identified by scanning degradome data and 5′-RACE [65]. MiR1428e-3p targets a sucrose non-fermenting-1-related kinase 1 (SnRK1b) gene, which has a regulating role in starch accumulation [20]. In addition, it has been reported that MYB and ACC, targeted by miR159 and miR819, regulates the phytohormone steady-state of ABA and ethylene in plants, respectively [21], [54]. Moreover, ABA concentration in the grains was positively correlated with the activities of SUS, AGPase, SS, and SBE [6], [53], while ethylene concentration was negatively related to the enzyme activities of SUS, AGPase, and SS [4], [66]. This indicates these miRNAs might have an impact on regulating the process of starch biosynthesis during rice grain filling (**Fig. 8**).

### Other miRNAs involved in regulating rice grain filling

More recently, Jeong et al [42] reported that simultaneously and panicle-preferentially expressed miR529-5p and miR156a-j downregulated SPL14, which regulated the panicle development by repressing panicle branching and consequently influenced the yield of rice [67], [68]. Although these miRNAs are important in panicle development, in our dataset, miR156a-j, instead of miR529-5p, was preferentially expressed during rice grain filling with a great abundance at middle and later stages of rice gain filling (**Fig. 7B**). Similarly, although ectopic expression of miR166 resulted in loss of HD-ZIPIII expression in the SAM region of the developing embryo [69] and was also found expressed in a development stage-dependent manner during rice grain filling (**Fig. 7D**). These together with the data of miRNA-regulated plant hormone homeostasis and starch biosynthesis suggest that two types of miRNAs are involved in coordinating the development of the panicle. MiR156 and miR166 play a role in overall spikelets development, while miR167, miR164, miR812, miR1861, miR1428 and miR45 play a specific role in the differentiation during rice grain filling (**Fig. 8**). In addition, four miRNA families (miR397, miR398, miR408, and miR528) may play a role in repressing their targets encoding copper-binding proteins and L-ascorbate oxidases in rice developing seeds as previously discussed for rice [25].

## Supporting Information

Figure S1 Plotting of novel miRNAs and their corresponding miRNA*s on the miRNA precursors.
(DOCX)

Figure S2 Plotting of miRNA and miRNA* sequences on the miRNA precursor loci.
(DOCX)

Figure S3 **Grain weight per kernel and grain filling rate during rice grain filling period.**
(TIF)

Table S1 **The primers used in this study.**
(DOCX)

Table S2 Predicted target fragments of miRNA*s from degradome data in starBase.
(DOCX)

Table S3 **Abundance (in TPM) of known miRNAs in each library.**
(XLSX)

Table S4 **known miRNAs greater than 10 TPM in at least one of our libraries.**
(XLSX)

Table S5 **Cluster of the known miRNAs (higher than 10 TPM) during rice grain filling.**
(XLSX)

Table S6 **Predicted targets of highly expressed known miRNAs.**
(XLSX)

Table S7 **KEGG pathway analyses of predicted miRNA targets.**
(XLSX)

Table S8 **Predicted targets of novel miRNAs.**
(XLSX)

Table S9 **Predicted target fragments of novel miRNAs from starBase.**
(DOCX)

Table S10 **Expression of miRNAs and their corresponding target genes during rice grain filling.**
(XLSX)
