# Supplementary material for: Characterization and Expression Patterns of microRNAs Involved in Rice Grain Filling
Source: PLoS One. 2013 Jan 24;8(1):e54148. doi: 10.1371/journal.pone.0054148 (PMC3554753; doi:10.1371/journal.pone.0054148)
Supplement: Figure S1 — Plotting of novel miRNAs and their corresponding miRNA*s on the miRNA precursors. (DOCX) [file pone.0054148.s001.docx]

**Figure S1.** **Plotting of novel miRNAs and their corresponding miRNA*s on the miRNA precursors.** (Five line for each novel miRNA alignment. The first line, miRNA name, location on the genome, length of the miRNA precursor, minimum free energy; the second line, miRNA precursor, miRNA precursor name; the third line, predicted stem-loop structure of the miRNA precursor, parenthesis and dot represent the base whether or not pairing with the ones at their opposite stem, respectively; the fourth and fifth line, the alignment of miRNA and their corresponding miRNA*s.)

> osa-miRn1 chr05|13105:22129892:22130035:+ 144(nt) -110.10(kcal/mol)

GGCGCCGGCGCGGCGATGTGGGGGAGGCGCGACGAGGGAGGGGTGGCGCGGCGATTTTTCGCAATACGGAGGGGCGCGCGGTGCGATGGTGGTGCGGACTACGGAGGAGGGTCGCGCCTCCCCCACATCGCCGCGCCGGCGCCC osa-MIRn1

(((((((((((((((((((((((((((((((((.........((.((((.(((.(((((((.....)))))))...))).)))).)).(((((....)))))........))))))))))))))))))))))))))))))))).

**********CGGCGATGTGGGGGAGGCGCG***************************************************************************************************************** osa-miRn1

******************************************************************************************************************CGCCTCCCCCACATCGCCGCG********* osa-miRn1*

> osa-miRn2 chr11|13111:20648228:20648347:- 120(nt) -29.60(kcal/mol)

CGGTCACTGTAACACACCGGATTCGAATCTTTTGCGTGAAAGCGAGGACGAAAAACGCTGTTAGATGTGGTTGGTTCAACTAAAAGAGAAGAGATGGATACCCGCGAGCGGCGGCGACAT osa-MIRn2

..(((.((((..(.(.(.(((((((..((((((((......))..((((.((...(((........))).)).)))).........))))))..))))).)).).).)..)))).)))..

**********AACACACCGGATTCGAATCTTTT*************************************************************************************** osa-miRn2

****************************************************************************************AAGAGATGGATACCCGCGAGCGGC******** osa-miRn2*

> osa-miRn3 chr11|13111:15903713:15903831:- 119(nt) -57.10(kcal/mol)

GGAGGTCTCGTCGACCACGTCGAAGAGGCTCACCTTGCGCTTCTGCTCCGTCCCCATCGCGGCTACGGTGGCGGCGAAGCGGGAGTCGGGTCGGTTCACGTGGTAAGGAGGAGACGGGT osa-MIRn3

....(((((.((.(((((((.(((..(((((..(((.((((((((((((((..((.....))..)))).))))..)))))).)))..)))))..))))))))))...)).)))))....

**********TCGACCACGTCGAAGAGGCTCA*************************************************************************************** osa-miRn3

****************************************************************************************GGTCGGTTCACGTGGTAAGGAGG******** osa-miRn3*

> osa-miRn4 chr07|13107:136856:136991:+ 136(nt) -82.29(kcal/mol)

TACAGGAATGTCAACTTCGTTTCGTGAGGCACCCACTATTCTGATAAATTCTACACCCACTAGGTTCTACCTCAAGTTTCTACATAATTTATCGGAATAATGGGTGCCTCACGAAACGAAGTTGACCTTCCTGTAG osa-MIRn4

((((((((.((((((((((((((((((((((((((.((((((((((((((........((((((.....)))..)))........)))))))))))))).)))))))))))))))))))))))))).)))))))).

**********TCAACTTCGTTTCGTGAGGCA********************************************************************************************************* osa-miRn4

**********************************************************************************************************CCTCACGAAACGAAGTTGACC********* osa-miRn4*

>osa-miRn5 chr02|13102:24114085:24114226:- 142(nt) -78.70(kcal/mol)

GGGCGCTGGGCGGTGACAGAAGAGAGTGAGCACACGGCCGGGCGGAACGGCACCGGCGGATGTGCCGTCGCGGCCGCGTGCTCACTGCTCTGTCTGTCATCCACTCCTCTCCCACCCCCCTCCACCTCCGGCCGGCGCGCCC osa-MIRn5

((((((.(((.((((((((((((((((((((((.((((((.(....(((((((........)))))))).)))))).))))))))).)))).))))))).)).)))...........((..((......))..)).))))))

**********CGGTGACAGAAGAGAGTGAGCAC************************************************************************************************************* osa-miRn5

*******************************************************************************GCTCACTGCTCTGTCTGTCATCCACT************************************* osa-miRn5*

>osa-miRn6 chr03|13103:18208040:18208382:+ 343(nt) -121.00(kcal/mol)

CCTACTCTTCTTAGTCTACGTTTCATCTCTCAACTCCAAAACTGGACAAAACATGTCCCTCATCTTCCCTAGCACTAGTTTAGAGTGGTTTTTGCCCATGTGGACTACTTGGCTAGTCGACTTGCATAACAAATGACATGTACAGGGTGAGCTAAGAGCCATGTCACATTTACCTCTCCTACTCCCTTTCTCTTCCACCTTCCCAAAAACTCCCCAGCAGACCTGGAGCGAAAATTGCCTTGAATTAGCTTCAGACCTATAGTGTCGTTGATAGTTTAGGGACATGTTTTATCCGGTATTGGAGTTGAGGGATGAAAATTGGACCAAGTCAAGAGTTTATGGA osa-MIRn6

((.(((((((((.(((((..(((((((((((((((((((.((((((.(((((((((((((...((((.......(((((((((.(..((((((((..((((((.((.((((((((.((.((.(((((.....))).)).))...)).).))))))))))))))).................................................((((.....)))).))))))))..).))))))))).....((((....).)))...)).))...))))))))))))).)))))).)))))))))))))))))))..))))).)))..))))))....)).

**********TTAGTCTACGTTTCATCTCTC************************************************************************************************************************************************************************************************************************************************************************************************************************ osa-miRn6

********************************************************************************************************************************************************************************************************************************************************************************************************************GGGATGAAAATTGGACCAAGT************** osa-miRn6*

>osa-miRn7 chr01|13101:23383482:23383570:+ 89(nt) -33.62(kcal/mol)

CACAAACTCCGTCCTCCCCACCATTGGTCAGCGGCGCACCGCTGCCACTGACAACACCTCTGCTAATGGTGGTCAGGACCAAATCTGCC osa-MIRn7

..........(((((..(((((((((((((((((....)))))).................)))))))))))..)))))..........

************CCTCCCCACCATTGGTCAGCG******************************************************** osa-miRn7*

**********************************************************TCTGCTAATGGTGGTCAGGAC********** osa-miRn7

> osa-miRn8 chr04|13104:7355271:7355494:+ 224(nt) -160.40(kcal/mol)

TAATTCGGTGCAATCATGCAACTCTCATCCTATATCGTTGGATCGAGAAATGGACATCCGAGATTCGTCCGCGTCACCATGAGTTAAAATTTAACTCACAGATTCACTCATGAGTTAAAATTTTCTCGGAGTTAAATTTTAACTCGTGGTGATGTGGACTAATCTCAGACGTCTATTTCTCGATCCAACGGTGTAGGATGAGAGTTGCATGGTTGCACCGAATT osa-MIRn8

.((((((((((((((((((((((((((((((((((((((((((((((((((((((.((.((((((.(((((((((((((((((((((((((((((((...((.....))..(((..........))).))))))))))))))))))))))))))))))).)))))).)).))))))))))))))))))))))))))))))))))))))))))))))))))))))

*************TCATGCAACTCTCATCCTATA********************************************************************************************************************************************************************************************** osa-miRn8*

*************************************************************************************************************************************************************************************************TAGGATGAGAGTTGCATGGTT********** osa-miRn8

> osa-miRn9 chr02|13102:27142265:27142413:+ 149(nt) -69.24(kcal/mol)

GGGCTTATCAAAGGGGCGCTTACTGAGAGTTCTTTGGCATTCTGTCCACCTCCTTGTCGAATCCTCAGAGACAGAAATCTCATATCTGTTGATCTTGGAGGTGGGCATACTGCCAATGGAGCTGTGTAGGCCTCCCTTTGTAAAACCCA osa-MIRn9

(((.....((((((((.((((((..(.((((((((((((.(.(((((((((((..(((((.......((((......)))).......)))))...))))))))))).).)))))).)))))).))))))).)))))))).....))).

**********AAGGGGCGCTTACTGAGAGTTCT******************************************************************************************************************** osa-miRn9

***********************************************************************************************************************AGCTGTGTAGGCCTCCCTTTG********* osa-miRn9*

> osa-miRn10 chr05|13105:16844628:16844715:- 88(nt) -52.20(kcal/mol)

ACGGCCCGAGTCTTATTGGGTCGTGCCTAGGCCGTTGGTGCAGTCCATGAGCGGGCATGGCACGACCCGGCATGTAGGTCGGGTCGTG osa-MIRn10

(((((((((.(..((((((((((((((..(.(((((.(((.....))).))))).)..)))))))))))..)))..).))))))))).

**********TCTTATTGGGTCGTGCCTAGGC******************************************************** osa-miRn10

*******************************************************CATGGCACGACCCGGCATGTAGGT********* osa-miRn10*

> osa-miRn11 chr09|13109:15791336:15791549:+ 214(nt) -86.84(kcal/mol)

TGGCATGCCATGTGTAGCCACATTGTAAGGGAGTCCTTGTGCACATGGTCAATCTCGCAGAAAGCCCACTGCAATCCAAATCTTCTTCACGATTGTTCCTATTCACCAAATCACCCGTCTTCTCGAATGACCGCCCACCATTTCCCTCTGTTGCTGTTGCTAACATCCATGGCCCAGGGACCCACTTGCAGCGTGGCTACATGTGGCATGCCAC osa-MIRn11

(((((((((((((((((((((.(((((((((.(((((((.((.((((((((.((..((((........))))..................((((..............))))............)).)))))................((((((....)).))))...))))).))))))))).))))))).))))))))))))))))))))).

**********TGTGTAGCCACATTGTAAGGG*************************************************************************************************************************************************************************************** osa-miRn11

***************************************************************************************************************************************************************************************ACTTGCAGCGTGGCTACATGTG********* osa-miRn11*

> osa-miRn12 chr11|13111:9050898:9051183:+ 286(nt) -78.70(kcal/mol)

AAACTCTTCCTGGCCTGTAGTTAGTAGAGGGTACTGCACAGAACATGCCAAGCAGAATATGAACTATACTTTGCGTTTGAAATGTATATGTGCATGCAAAAACACATAGTTTATGGAGTGCTCTTCTTGCTTCCCGCATACCTTCCCAATAACACCTTCCCCGTTCACATCAGAAACAACCCGGTCTCTCCATCGGATGATCCAGTGGCGCGAGTAAAGCTGGCACGAGGAATAGTACACACCACCTAGCATGCATGCCTCTGGAACTGACAGGCGAATGGAGTAT osa-MIRn12

..(((((...(.((((((((((..((((((............((((..((.(((((.((((...)))).)))))...))..))))....((((((((..((((.....)))).(((.(((..((...(((.....)))..................((((.(((................(((((......))))).....(((((.((....))...))))).))).)))).))..))).)))....)))))))).)))))).)))).)))))).)..)))))..

**********TGGCCTGTAGTTAGTAGAGGG*************************************************************************************************************************************************************************************************************************************************************** osa-miRn12

******************************************************************************************************************************************************************************************************************************************************************CTCTGGAACTGACAGGCGAAT******* osa-miRn12*

> osa-miRn13 chr06|13106:21844755:21844887:- 133(nt) -81.30(kcal/mol)

CGCACGGCTTAAAATAATGTATCTGTGCTGACTAGGCACTAGGGGGCCCGATATGTGCATTTCGGTCACTTCGCGTAAAATATCGGATCCCCTAGCACCTCGTCAGCACGGATACATTATTTTAAGCCGCGTA osa-MIRn13

.((.((((((((((((((((((((((((((((.(((..(((((((..(((((((.(((....(((.....))).)))..)))))))..)))))))..))).)))))))))))))))))))))))))))).)).

************AATAATGTATCTGTGCTGACT**************************************************************************************************** osa-miRn13*

******************************************************************************************************TCAGCACGGATACATTATTTT********** osa-miRn13

> osa-miRn14 chr06|13106:29361479:29361817:- 339(nt) -92.40(kcal/mol)

AGGCATCGTTAACAGATAGAGAATCTTCTCAGAAAATTCTCCAAATTTGATGTTTGAAATCTGACTGATTCTTTGAGAACTGTGAGCCTGTAGTTAAGCTATCAGCTTGAAATCCTTTACTGCAGCACTACATACTCCGGAACCTTGTTACTGTTATTAGCCACCAGCAAGCCAGCTCAATTTACAGTTAGTGCCACCACAAAAGGACTCTTCCCAGTTATATGTAAGCTATACCACCCAGATATTCAGCTACACCTTGAAGGCATAAGAAGCATATGCTTTTGGATACAATCAAATGGAGAATCTGATGGGAATATTCTTTATCTGTTAACAACAAAC osa-MIRn14

.......(((((((((((((((((.((((((..(.((((((((..((((((.........(((((((.....(((((..(((.(((..((((((...(((..(((..((((....))))))).)))))))))..))))))...((((((..((........))..))))))....)))))....))))))).(((.........(((....)))........((((.((((((.(.....).)))..))))))).......)))...((((((....)))))).......)))))))))))))).)..)))))).))))))))))))))))).......

************CAGATAGAGAATCTTCTCAGA****************************************************************************************************************************************************************************************************************************************************************************************************************** osa-miRn14*

********************************************************************************************************************************************************************************************************************************************************************************************************************TGGGAATATTCTTTATCTGTT********** osa-miRn14

> osa-miRn15 chr09|13109:10835586:10835832:+ 247(nt) -120.20(kcal/mol)

TGCTTCTTGTTAAATGGAGAGAACGAAAGAGAGATAGGGTCCTCTGCAATACTCACCTGAGCCTATGGCAGCTCCAACTCTCAGATTCACCTCAGTTCTGACTAGAGCGCAACCAAACGGTCCAGCTCCACAAAAAAATGGAGTGGAGTAGGCCAGATCGCTCCAAAAAATTGTACTAGCTGCGCTCCCAACTCCAACTCCAGAGGACCCTATCTCTCTTTCGTTCTCTCCCTTTAACAAGAAGCAT osa-MIRn15

((((((((((((((.(((((((((((((((((((((((((((((((.....(((....)))....(((.((.........(((((...........)))))...((((((........((.((.(((((((...........))))))).)))).....(((.(((....)))....))).))))))....)))))....))))))))))))))))))))))))))))))).)))))))))))))).

**********TAAATGGAGAGAACGAAAGAG************************************************************************************************************************************************************************************************************************ osa-miRn15

*************************************************************************************************************************************************************************************************************************CTTTCGTTCTCTCCCTTTAAC********* osa-miRn15*

> osa-miRn16 chr10|13110:3929128:3929337:+ 210(nt) -141.90(kcal/mol)

GGAGGGGGAGCCGAGTGCCGTTGCGAGTCTGACCTCCACCTCGTTGTGCTCGGCCGCAACCACGGCTTTCAATCGGCCGACGACGGCGTCCCTCCCTCCTCGGCCATCCACTGACAACGCGGACGGCTCCGCGGATCTTGGCGAATCCTGCGGTTGCAGCTGAGCGCGGTGAAGCGAGGTCGGACTCTCGGCGGCGCTCGGCTCCCCCTC osa-MIRn16

.((((((((((((((((((((((.((((((((((((..(.(((..(((((((((.((((((.(((..(((....((((((.((.((.(.....))))).))))))..(((..((...((((((....))))))..)).))).)))..))).)))))).)))))))))..))).).)))))))))))).))))))))))))))))))))))

*************AGTGCCGTTGCGAGTCTGACC******************************************************************************************************************************************************************************** osa-miRn16*

***********************************************************************************************************************************************************************************TCGGACTCTCGGCGGCGCTCG********** osa-miRn16

> osa-miRn17 chr12|13112:4864157:4864478:+ 322(nt) -115.80(kcal/mol)

TTTTCCTACATCTTTCACATGGTATTAGAGCTGAATTCTAAACCGTTGAATGATCGTCCAACAGCAACCCACGGCTACTAGGAGCTGTAGCTCCCACGGTAGTACCAAATCTGTTGCTTTTTCTGATCTGTTTCTTGTCATGAAAATCACTTTGAGATCTGTAGTGGAGGCTACATGGCATTGTTTTCCCCAAGTCTGTGGCTATCAACAGATTTGTGACCTGGCGTTGCTGCCATAATCAGCAGTGTGTTTGTGTTGGCTCTGCTTGGGGTTTAGCATCTTAGGTGTTAGCTCTGATACCTTGTGAGAGATTTAGAGACTT osa-MIRn17

...(((((.((((((((((.((((((((((((((..(((((...(((((((.....((((.((((((((((..((((((.(((((....)))))...))))))..(((((((((((..(((((((((.........)))).))))).((((..(....)...))))..(((((((.(((.(((.......)))))))))))))..))))))))))).....))).)))))))........(((((...(((......))).))))))))))))))))...)))))..)))))))))))))).)))))))))).))).))...

**********TCTTTCACATGGTATTAGAGCT************************************************************************************************************************************************************************************************************************************************************************************************** osa-miRn17

***************************************************************************************************************************************************************************************************************************************************************************************************CTCTGATACCTTGTGAGAGATT********* osa-miRn17*

> osa-miRn18 chr01|13101:7445343:7445423:- 81(nt) -35.40(kcal/mol)

CAAAAAAACCCCCCAATCCTAGGGATGTATCTGGACAACGTGTATGTCTAGATATATATCTAGGATTGGATTTTTTTTTTT osa-MIRn18

.(((((((....((((((((((..(((((((((((((.......)))))))))))))..))))))))))....))))))).

************CCAATCCTAGGGATGTATCTGGA********************************************** osa-miRn18*

************************************************TAGATATATATCTAGGATTGGAT********** osa-miRn18

> osa-miRn19 chr03|13103:34000873:34001018:+ 146(nt) -72.20(kcal/mol)

GTCATTATTCTTCAGTCCGTTGTCCTTTGTGATAACATATCTGTACTTATACGTTTGTTCATTTAGGATAGACAAGGCTCAAAGCCACGCTAGCTGACATTAGCCGGATATGTTATAACAAAGGACAACAGACTGAAGAATAATGA osa-MIRn19

.((((((((((((((((.((((((((((((.((((((((((((.........((((((((.....))))))))..(((.....)))..(((((......))))))))))))))))).)))))))))))).))))))))))))))))

*************AGTCCGTTGTCCTTTGTGATA**************************************************************************************************************** osa-miRn19*

*******************************************************************************************************************TAACAAAGGACAACAGACTGA********** osa-miRn19

> osa-miRn20 chr11|13111:5399585:5399707:+ 123(nt) -49.60(kcal/mol)

GAGACCAAGTCTCTGTTTACACCGTCCTCAAAACATGACAGGTAGACATAACTGCCATCTACAAGTAGTTAATTCTAGAGTACGTTAGTGCTTTGAGTAGGGTCTAAACAGAGACCTGGTCTA osa-MIRn20

.((((((.(((((((((((.(((.(.((((((.((((((.(((((......))))).((((.((........)).))))....)))).)).)))))).).))).))))))))))).)))))).

************CTGTTTACACCGTCCTCAAAACA**************************************************************************************** osa-miRn20*

******************************************************************************************CTTTGAGTAGGGTCTAAACAGAG********** osa-miRn20

> osa-miRn21 chr07|13107:11010275:11010501:+ 227(nt) -70.20(kcal/mol)

AAGGGCCAGGCAGGCAGAGCATGAAGAGCATCCTCCGAGGAGGTGACAAAATGGAGGTTGAGCTTCCTTAGCTTCGGGAAATTAATGGAGTAGCTGGCTGATGATGTCATCATGTAAGCGGCAGCTTGAGAAGCTAACCACCTCCAAGCTAGGTAGAGGAGAAGTGAAGTGCATATATTACTAGCAAGTAGTTTATGTCATTTGCCTGATCAGTCATCGTGGTGTTG osa-MIRn21

.((.((((.((((((((((((((((..((...((((..(((((((.......(((((.....))))).(((((((..........(.((((.(((((((((((....))))).))...)))).)))).))))))))..)))))))...((....)).))))..((..((((.......)))).))..))..)))))))..)))))))).........).)))).)).

**********CAGGCAGAGCATGAAGAGCAT**************************************************************************************************************************************************************************************************** osa-miRn21

*******************************************************************************************************************************************************************************************GTAGTTTATGTCATTTGCCTGAT***************** osa-miRn21*

> osa-miRn22 chr04|13104:4406337:4406583:+ 247(nt) -84.50(kcal/mol)

GAAGAATGATCTGATTGAGCCTCGTCAATTCTCCCTCTGGTTCCAAATGTTGGTCGTTTTAGAGCTGTGCACAGGAACTAAGAAGTGTTACAATTATCTTGTTGCCCTGCAATTATTGCAGATCAGTAGTGATACTTCATTTATTCATGAGAATCGTCGCATTCAATTATCAAGGGTGTCTGGGAAAAATGGGGTTCAATTGCCCCTGAAACAACCTACATTTGGAACCAGAGGGACTGTCGTTTTT osa-MIRn22

.(((((((((..((((((......)))))).(((((((((((((((((((.(((.(((((((.(..(.(((...(((((..((((((((((.(((((((.(((((...)))))......)))).))).)))))))))).......(((.....(((..((((((.........)))))).))).....))).)))))...)))))))))))).))).)))))))))))))))))))..)))))))))

************************************************************************************************************************************************************************************************************************TACATTTGGAACCAGAGGGAC********** osa-miRn22

> osa-miRn23 chr02|13102:18552430:18552643:- 214(nt) -113.10(kcal/mol)

GGGCCTTCCATGAGGACAAGAGCTGATTCGGTAGCCAAGGATGACTTGCCTAATGCCTATGTGCATGTGTTTATACGCTGCTCATCTGCATTTTGATTATCCCCTGATCAGTCCTGTCGTCAATTATATGTGTGTGTAGTACTCTGTACTCATACATATATAGGCATGTCTTCCTTGGCTATTCGGAGCGGCTCTTGTCTCTCGTGGAAGGCTG osa-MIRn23

.((((((((((((((((((((((((.(((((((((((((((.(((.((((.(((((..(((.(((.((((....))))))).)))..)))))(((((((.....))))))).............((((((((((((.(((((...))))))))))))))))))))).))).)))))))))).))))).))))))))))).))))))))))))).

**********TGAGGACAAGAGCTGATTCGG*************************************************************************************************************************************************************************************** osa-miRn23

> osa-miRn24 chr02|13102:18968871:18969037:- 167(nt) -68.00(kcal/mol)

GAAATACATTCATACCAAATCTCAAGTCCAAACTTAGCTTCATTTGAGAGAAACAAAAAAGAGAAATTCTAGGTGAATAGTGTCATGTTACCATTCACCCAAAATGTCTTTTCTGTTACTCCTAAATAAAGTTGAGTTTGAACTTGATATTTGGTGGGAATGTATTT osa-MIRn24

.((((((((((.((((((((.((((((.((((((((((((.(((((.(((.((((.((((((...(((...(((((((.(((......))).)))))))...))).)))))).)))).))).))))).)))))))))))).)))))).)))))))).))))))))))

****************************************************************************************************************************************TTTGAACTTGATATTTGGTGG********** osa-miRn24

> osa-miRn25 chr03|13103:15638443:15638532:+ 90(nt) -38.21(kcal/mol)

TTAGGGTATTAATGTATTATGGTCTTTTCTCCTCTCTCTCGTATAAAATATATTTTGTACGAGAGATGGGAAAAGACAACAATACCCCTA osa-MIRn25

.(((((.......(((((...((((((((.((..((((((((((((((....)))))))))))))).))))))))))...))))))))))

*********************************************************TACGAGAGATGGGAAAAGACAAC********** osa-miRn25

> osa-miRn26 chr03|13103:1656121:1656461:- 341(nt) -87.94(kcal/mol)

GGAGATTGCATTTCTTGACTCGGGATGACTAGATATTGCTGACGTCACTAACTTTGTTCATCCTGGTACTCCTCTTGATGAGGAGGCCTCACAAAGGGGCACTTCTGTATATCTTGTTGGACAGCGAATTGACATGCTTCCAAAGCCTCTTACTGAAGGTATCTCTATTACTTATGTCGTATGCACACTGGATTAACCCAAAGCGGAACGTTTCTATTTCAATTTAAGAATACTCATATAATGTTAAAGTATTAGTGAAAGTAACTAATGATCTTCTCAAATATGCTGGTTTCTAGATGTTTGTTCTCTTCGTGCTGATGTTGAGAGATTGGCGTTCTCTG osa-MIRn26

(((((.(((((((((..((((((.((((..(((..(((((....((((((((((((..(((...(((.((((((.....)))))))))......(((((..((((.(((.......(((((.((((........))))))))).......))).))))...)))))......((((..((((.(....(((......)))....((((........)))).......).)))).))))..))).))))))..)))))).))))).............(((((((.((((...))))))))))).)))..)))).)))).))..))))))..))).))))).

**********TTTCTTGACTCGGGATGACTAG********************************************************************************************************************************************************************************************************************************************************************************************************************* osa-miRn26

> osa-miRn27 chr03|13103:5370801:5370924:- 124(nt) -45.70(kcal/mol)

AGTTGAGGGGTCGGGATATGTGGTATTGCGGTTGAAATAAGTTCATTTGAGATCCCTTAACTTGACAGCAAATTCAATTTTTATCTTTGAACCGCAATACCAGATACAATGGGTCCTTCAACTA osa-MIRn27

(((((((((..(.(..(((.(((((((((((((.(((.........(((((....))))).((((........))))........))).))))))))))))).)))...).)..))))))))).

**********TCGGGATATGTGGTATTGCGGTT******************************************************************************************* osa-miRn27

> osa-miRn28 chr04|13104:20478461:20478783:- 323(nt) -153.80(kcal/mol)

CGTGCGGTCTCACGGAATGGAAGAGCGAGACCGTTCGGTCTCGCAGAAGCAATCAGCGACATTGTCCAATAGGATCCATTTGATTAGTGGTTAATTACTTAATTAACATTTAACTAATAATAATTAGTGATTAATTATTCTAATTAATGATTAATTAATTAATTATTAATTAATCACTAATAAGGATAATTAACCACTAATTAAATTGGCAAGGCACGGTATCGCTCTTTGCTTGAGCGATAGCGTTGATTGCTTCAGCGCTATCGCTGTTTGCTTGGGCGAGACCGCGCGGTCTCGCACTGCTACCGTGCGAGACCGTGCGC osa-MIRn28

((..(((((((((((..(((.((.((((((((((.(((((((((..((((((.((((((..(((.(((((............((((((.(((((((....))))))).....))))))..(((((((((.(((((((((((.((((.((((((((((((.....)))))))))))).)))).))))))))))).))))))))).)))))))).......((((((((.......))))))))(((((((.....)))))))..)))))).))))))..))))))))).)))))))))).)).)))))))..)))))))..)).

**********CACGGAATGGAAGAGCGAGAC**************************************************************************************************************************************************************************************************************************************************************************************************** osa-miRn28

> osa-miRn29 chr05|13105:811715:811906:+ 192(nt) -115.60(kcal/mol)

GCGCCGTCGCACCCCCCTTTGCCGGCGCGCGCACTCACCCGAGAGTGAGTGCGCCGCCGTCGAGCGGCTGTGCAGCGGCGCCCGGCAAGCCGGCGGCGCGGCGCCGCCCCCTCTTTTCTCTCCCGCGTCGGCGTGAGGACGACCGGTGGTGGCATGACTCCCGCGCGCGTCGGCGGCGGCCGGCGGCGGCGG osa-MIRn29

(((((((.((((..((.((((.(((((.((((((((((......))))))))))))))).)))).))..)))).)))))))(((((..(((((((((((((.((((((.((..((.((.(((.(((....))).))))).))..)).)))))).......)))))).)))))))....))))).........

**********ACCCCCCTTTGCCGGCGCGCGC**************************************************************************************************************************************************************** osa-miRn29

> osa-miRn30 chr05|13105:26212578:26212696:+ 119(nt) -61.60(kcal/mol)

TGTGATGTGATGAAAAGTTGGGAATTTGGAGGAAGATTGATGTGAACTAAACAGGGCCCCAATCCACGTTTAGTTCACACACAACTTCCCCCAATCTCCCAACTTTCCATCGCATCTCC osa-MIRn30

...(((((((((.((((((((((..((((.(((((.(((.((((((((((((.(((......)))..))))))))))))..)))))))).))))..)))))))))).)))))))))...

**********TGAAAAGTTGGGAATTTGGAG**************************************************************************************** osa-miRn30

> osa-miRn31 chr05|13105:22710968:22711219:- 252(nt) -94.30(kcal/mol)

ATCTAGGGTTTGGGTTTGGGTGGTGGTGGTGCAGGAGGGGGGAGGTGGGATGGCGGATGGGCCGGGGAGCCCGGGGGGAGGAGGGGGGAGCCACGAGAGCGGGAGCCCGAGGGGGGGAGGGGGAGGAGGGGGAGGTGGGGGTGGGGGTGGGGGGGTGAGGGAGCAGGACAGGTTCCTCCCCATCGCCAACATCAGCCGCATCATGAAGAAGGCCATCCCGGCCAACGGGAAGATCGCCAAGGACGCCAAGGA osa-MIRn31

........(((((((((...(((((((..(.(....(..((....(((((((((.......((.(((..((((...........((....)).(....)))))..)))..))...................((.((((.((((..(((((..(((.((((((.(......).))))))))).)))))..))))..)))).)).........))))))))).))..).).)..))))))).)))).)))))..

**********TGGGTTTGGGTGGTGGTGGTGCA*************************************************************************************************************************************************************************************************************************** osa-miRn31

> osa-miRn32 chr06|13106:637204:637356:- 153(nt) -69.70(kcal/mol)

GCTATTGAACTAGATGGCTGATCTGGTGTGGCTTGGCTTTGGGTAAGAAGGATTGGCCTCTGTCTAGTGCCTCACTTTCCTTCTGCGGTTCTGTCTACTGTCCTGGTTTGGAAGAAGTCAAACCAAGCCAGATCAGCCATCTAGTTGAGTCTC osa-MIRn32

...(((.((((((((((((((((((((.(((.(((((((((..(.(((((((..(((((......)).)))......)))))))...)..).........(((......))).)))))))).))).)))))))))))))))))))).)))...

**********TAGATGGCTGATCTGGTGTGG************************************************************************************************************************** osa-miRn32

> osa-miRn33 chr06|13106:4371633:4371745:- 113(nt) -62.40(kcal/mol)

TACTCCCTCCGTCCTAAAATAAACCAACCCCGTACGGGATGTGACACATCCTAGTACGATGAATCTGGACACACATCTGGTATGAGATTGGTTTATTTTGGGATGGAGGGAGT osa-MIRn33

.((((((((((((((((((((((((((.(.(((((.(((((((.....(((.((..........)))))..))))))).))))).).))))))))))))))))))))))))))

**********************************************************************************TGAGATTGGTTTATTTTGGGA********** osa-miRn33

> osa-miRn34 chr06|13106:9231124:9231271:- 148(nt) -82.10(kcal/mol)

TTCCATGCGGTTCGTAAGTGGAACCGCACGGGAAAATAGGGGGACCGGTGCCGGATTATCGCCCGCACGGGAAAATACATTTTCCTATGCGGGCGTCTTAATCCGATTCATTATCCCGTGCGGTTGATTTAAGAGGACCGCACAGAAA osa-miRn34

(((..((((((((.(((((.((.((((((((((.(((.((....)).....(((((((.((((((((..((((((....))))))..))))))))...)))))))....))).)))))))))))).)))))...))))))))..))).

**********TTCGTAAGTGGAACCGCACGG********************************************************************************************************************* osa-miRn34

> osa-miRn35 chr08|13108:5718281:5718494:- 214(nt) -85.50(kcal/mol)

GTCTATTTTCCCTCCCTCATCTCTTACTCTGATTGAATCGTCTCTGTTATGTGCAAAACCAGGTATAACGTCTCCCCATCTTCAAAAAATGGTAAAAAATCACTGAAGTAGTCGATTTTCACCGGTTTTCAAAGAAGAGATAGTCATTATACCTGGTTTTAAGGTTGAGGTAGACGAACCAATCAGGAGCAGAAGATGAGGGAGGCAAAATAGA osa-MIRn35

.((((((((.((((((((((((((...((((((((..((((((((...((.(..((((((((((((((((((((....((((..((((.((((..((((((((((...)))).)))))).)))).))))..)))).))))).))...))))))))))))).).))...)).))))))..))))))))...)).)))))))))))).))))))))

**************************************************************************************************************************************************************************************TCAGGAGCAGAAGATGAGGGAG********** osa-miRn35

> osa-miRn36 chr09|13109:15610785:15611045:+ 261(nt) -96.00(kcal/mol)

TACTTAATCTTCTCACTTTGGACTAGGTATTTTTGTCATTTTTTCATTTTGGACCACTCAAACTCTCTTCGTGATCACATCAACAGCCTCAAGCACATCGGCACCCTCATTCCGATGGTCTCCCCTTTTACGTTCAGCTCACATCTTTGCTGGAGGAGAGGTTGGCTAGAAAAAAGAGTTAGGGTGGTCCAAAGTGAAACAAAGGGGAAAAAAAACCTAGTCCAAAGTAAAAAAGATTGGATAGAGGGTGGTCGGAAGTGA osa-MIRn36

(((((((((((...((((((((((((((.(((((.((.((.((((((((((((((((((.((((((.(((.((((...)))).(((((((..((.((((((..........))))))))...........(.((((((..........)))))).).)))))))....)))...)))))).)))))))))))))))))).)).)).)))))...))))))))))))))....)))))).(((........)))..))))).

**********TCTCACTTTGGACTAGGTATT************************************************************************************************************************************************************************************************************************************** osa-miRn36

> osa-miRn37 chr09|13109:16039504:16039708:- 205(nt) -80.96(kcal/mol)

TGTTTGGTTTGAAGCCAATTTTTGTCCTACCAAAATATTGGTAGTGCCAAAACCTTGGCAAGTTTTGGGCCTTTTGGCACTACCAAATTTTGTAGTATTGACTCTGAACTATTATAGGCAAAATTTGGTACCAAACCAAATGTACATTTACCACTATTAAAGTTACCAAAATTTTGGTAAGGCGAAAATTGGCATCAAACCAAAC osa-MIRn37

.(((((((((((.(((((((((((((.(((((((((.((((((.((((((....))))))...((((((((....)))..(((((((((((((..(((...............))).))))))))))))).....))))).......................)))))).))))))))).))))))))))))).)))))))))))

*****************************************************************************************************************************************************************************TTGGTAAGGCGAAAATTGGCAT********** osa-miRn37

> osa-miRn38 chr10|13110:11792586:11792675:+ 90(nt) -60.80(kcal/mol)

ACGTGCAGCAGGACGATCAACGCGTTCCCCAATTATCTTCGACACTTCTTGTGTGATAATTGGGGAACGCGCCGATCGTCATGCTGTACG osa-MIRn38

.(((((((((.(((((((..(((((((((((((((((....((((.....)))))))))))))))))))))..))))))).)))))))))

***********************************************************TTGGGGAACGCGCCGATCGTC********** osa-miRn38

> osa-miRn39 chr12|13112:1969860:1970197:+ 338(nt) -106.00(kcal/mol)

AATTGCATAATGCTCCGGATATTATGGCATGTAGCATATAACCCTAGTCATTATGGTATCAAGGTTTCGCAAAACCCAGCATTCACTCAAATCATAAAAAAAACAACTTGTTGACACATATTCACTTCATTTTTCATGCCATAAACCACTATGTATTGTTCAAATGTTGGTTACTTGATCAGGATGACCAGTTTAATCAGTCAACGTTTTGAACATTATTATGTCAAGGATGGTAAAATTAGCTGAAATTTGAACTAGGATGGATTTTATGATACTTCTAGGTTATAGAGCATAGGGTTATGTGCCTCGAGTCATAATATCCGGAGGTTTATGCAACT osa-MIRn39

..((((((((..((((((((((((((((.((..(((((((((((((........(((((((.(((((....)))))...(((((...................(((....)))............((((..(((((((((((.....((...(((.(((((((((((((..(..((((...((....))...))))..)..))))).)))))))).)))...)).....))))))........)))))..))))...))))).......)))))))((((.....))))...)))))))))))))..)).))))))))))))))))..))))))))..

**********TGCTCCGGATATTATGGCATG******************************************************************************************************************************************************************************************************************************************************************************************************************* osa-miRn39

> osa-miRn40 chr12|13112:7986915:7987137:- 223(nt) -50.60(kcal/mol)

ATGAGGTCCGAACATAGCTCCTCAAATTTGGCTCTGGAGAGAGTTGTCTCGATGTGTTTAGGCCCATCAGCAGTAGCAGTTATGAAAGGCAAGCTGCAATTCAAGACATTCAATTATTTAAGAAGAAAAATCACCAAATGAGCAATAGAATACTCTGTGAGAACAATTGGAAAGAAGCTCACCTAATGTTTGTCTGAGACAGCGTAGACAGTTCCATCTTCGC osa-MIRn40

.(((((..(.......)..)))))...........(((((((..(((((((.(((.(((((((.(((.((..(((((.(((.((((..((.....))..)))).)))..........................((((.((..(.(((((....)))))..)..)).))))......))).)))).)))...)))))))))).)).)))))..))..)))))..

************************************************************************************************************************************************************************************************TCTGAGACAGCGTAGACAGTT********** osa-miRn40

> osa-miRn41 chr04|13104:20638203:20638329:- 127(nt) -73.10(kcal/mol)

GATCAGATTGCATCGGCATCTCTACTCTGCAGCCGCTGCCTCTGCCGCCTCTTTCATCCGCTGTCCGTGCGACGGCAAGAGAGTGTTGACAGCGGCTGCGAAGTAGAGATGCCGACTTGATCTGAGC osa-MIRn41

..((((((((..((((((((((((((.(((((((((((..((...((((((((......((((((.....))))))))))).)))..))))))))))))).))))))))))))))..))))))))..

************************************************************************************************TGCGAAGTAGAGATGCCGACT********** osa-miRn41

> osa-miRn42 chr09|13109:20111005:20111192:+ 188(nt) -52.72(kcal/mol)

GGTGATCAGCATCTCGATGGTGATTGTTGCTAGGCGTAATCATACTATACCTTCTCTGCAAAGGAATTTCCCTTTCATCGTAGTAAGGTTGTGATTCTGGTGCAGTTAGTTGTGGCCTTTGTACAGCAGGAGTGGGAGCAGAAGCAGGAGCAGCAGCAGACATTTGCGGAATGTGTAATACTACACCG osa-MIRn42

((((....((((..((..((((.((((((((..((..............(((.((((((.((((......)))).....(((..(((((..((((((((...)))..)))))..)))))..))).))).))).))).((....))....)))))))))).))))..))..)))).........)))).

**********ATCTCGATGGTGATTGTTGCT************************************************************************************************************************************************************* osa-miRn42

> osa-miRn43 chr10|13110:6746076:6746192:+ 117(nt) -54.24(kcal/mol)

AGAGAATTTATTATTTGTCATTCTTCCAAAATACACATGTCTAAATGAAAACGAAAATTTAGAAGAGTAGTATTTGGACATGTGAATTTTGGGAGGATGGCAAATAGTAAATTATCC osa-MIRn43

.((.((((((((((((((((((((((((((((.((((((((((((((...((..............))..)))))))))))))).)))))))))))))))))))))))))))).)).

**************************************************************************************TTTTGGGAGGATGGCAAATAG********** osa-miRn43

>　osa-miRn44 chr11|13111:27423178:27423250:- 73(nt) -32.10(kcal/mol)

TAGTCGCCGCCGGGGTCGTGGGAGAGGCGGTGCGCGAAGTCGTCGATGCAGTCCTCGATGTCGTAGGTGACGT osa-MIRn44

..((((((..(((.((((.((((...(((...((((....)).)).)))..)))))))).)))..))))))..

******************************************TCGATGCAGTCCTCGATGTCG********** osa-miRn44

＞　osa-miRn45 chr03|13103:13022501:13022783:+ 283(nt) -66.49(kcal/mol)

AGGACAGGTAGCTGGAGTAGCTCAGATGGTTAGAGCGTGTGGCTGTTAACCACAAGGTCGAAGGTTCAAGCCCTTCCTCTAGCGATATTTTTTTAAATTTTTTTTAGACTTTTCTGCTTTGCTGTTTGAACAATTTGTGTGAGAGAATTTGACCATTTGTTGCATTGCTGTTTGAACAATTTATATATGTGATAATTTCAACAATTTCTGCTTTGTTGATATTTTTTATAATTTTTTTAGACTTTTCTGCTTTGCTGTTTGAACTTTTCTGCTTTGCTGTTTG osa-MIRn45

.((((((..(((.((((.((.(((((((((.(((((.(((((.......))))).((((....((((((((..........((((((..(..((((((((((((.(.((..(((.((......))..)))......)).)))))))))))))..)..))))))......))))))))..........(((((.(((.(((((((........))))))).)))..))))).........)))).....)))))))))))))).)).)))))))...)))))).

**********GCTGGAGTAGCTCAGATGGT************************************************************************************************************************************************************************************************************************************************************* osa-miRn45
