# Supplementary material for: Characterization and Expression Patterns of microRNAs Involved in Rice Grain Filling
Source: PLoS One. 2013 Jan 24;8(1):e54148. doi: 10.1371/journal.pone.0054148 (PMC3554753; doi:10.1371/journal.pone.0054148)
Supplement: Figure S2 — Plotting of miRNA and miRNA* sequences on the miRNA precursor loci. (DOCX) [file pone.0054148.s002.docx]

**Figure S2. Plotting of miRNA and miRNA* sequences on the miRNA precursor loci.** [Five line for each miRNA precursor alignment. The first line, miRNA precursor name; the second line, miRNA precursor, miRNA precursor name, length of the miRNA precursor; the third line,predicted stem-loop structure of the miRNA precursor (parenthesis and dot represent the base whether or not pairing with the ones at their opposite stem, respectively), minimum free energy; the fourth and fifth line, the alignment of miRNA and their corresponding miRNA*s.]

>osa-MIR1425

CTGTTGACTGCATTAGGATTCAATCCTTGCTGCTAAATGTATTGCTTATATTCAGCAATATAATGTTCAGCAGCAAGAACTGGATCTTAATATAGTCGATAG osa-MIR1425 102

((((((((((.(((((((((((...(((((((((((.(((((((((.......)))))))))...)).)))))))))...))))))))))).)))))))))) structure -46.00

*************TAGGATTCAATCCTTGCTGCT******************************************************************** osa-miR1425

**********************************************************************CAGCAAGAACTGGATCTTAAT*********** osa-miR1425*

>osa-MIR1433

AGGCCATCTTCGATAGCCAAGGATGATTTGCCTGTAGCCCCATTGTCATCAGCTCTCTCCGTCGAGAGCGACAACCGGGCTCTACTGGCAAGTCTCCTCGGCTACCCGAGTACCTCTTATGCTATCCCA osa-MIR1433 129

(((....(((.(.(((((.((((.((((((((.(((((((..(((((....((((((......)))))))))))..)))))..)).)))))))))))).))))).).)))..))).............. structure -53.60

*************************************************************************************TGGCAAGTCTCCTCGGCTACC*********************** osa-miR1433

*************TAGCCAAGGATGATTTGCCTGT********************************************************************************************** osa-miR1433*

>osa-MIR1884b

TCTGTTTTTAAATGTATGACGCTGTTGACTTTTAGATATATGTTGACAATTCGTCTTATTCAAAAAATCGAGCAAATATAAAAATATAAGTGAGGCTTAAGGTATATTTGATGATAAAACAAGTCATGGCAAAATTAATGATACCTGTATAACTTTTTTGAGTAAGACGAGTGGTCAAACGTAGATTTAAAAGTCAACGGTGTCATATATTTATAAATGGA osa-MIR1884b 221

(((((((.(((((((((((((((((((((((((((((.(((((((((.(((((((((((((((((((.....((((((((....(.((((.....)))).).))))))))........(((.(((((..........)))))...))).....))))))))))))))))))).))).)))))).))))))))))))))))))))))))))))).))))))) structure -86.30

**********AATGTATGACGCTGTTGACTTTTA******************************************************************************************************************************************************************************************* osa-miR1884b

*********************************************************************************************************************************************************************************************AAAGTCAACGGTGTCATATATTTA******** osa-miR1884b*

>osa-MIR408

GGGAGTTCTGTGATTGGAGAGGAGAGGAGACAGGGATGAGGCAGAGCATGGGATGGGGCTATCAACAGATGTAGATTATTCCTTGCACAAGAGATGATGATGAGCTGTGAATGAGTTCTGAGAGATGGCTGGTGTTGTTGTTGCTCCCTCCCCTGCACTGCCTCTTCCCTGGCTCCCCTGCACACCTCTCTCTCTCTCTCTCTCTCTCTGTGT osa-MIR408 213

(((((....(.((..((((((((((((...((((((.(((((((.(((.((((.(((((...((((((......(((((..(((....)))..))))).((.((((((...(.(....).)...)))))).)).))))))..))))).)))).))).))))))).))))))((......))...)))))).)))))).)).).)))))..... structure -90.00

********************************************************************************************************************************************************CTGCACTGCCTCTTCCCTGGC**************************************** osa-miR408

******************************CAGGGATGAGGCAGAGCATGG****************************************************************************************************************************************************************** osa-miR408*
