# Supplementary material for: Characterization and Expression Patterns of microRNAs Involved in Rice Grain Filling
Source: PLoS One. 2013 Jan 24;8(1):e54148. doi: 10.1371/journal.pone.0054148 (PMC3554753; doi:10.1371/journal.pone.0054148)
Supplement: Table S1 — The primers used in this study. (DOCX) [file pone.0054148.s004.docx]

**Table S1.** **The primers used in this study.**

| **Primers for QPCR** | |
| --- | --- |
| miR159a.1,b_RT | CTCAACTGGTGTCGTGGAGTCGGCAATTCAGTTGAGCAGAGCTC |
| miR159a.1,b_F | ACACTCCAGCTGGGTTTGGATTGAAGGGA |
| miR166a-d,f_RT | CTCAACTGGTGTCGTGGAGTCGGCAATTCAGTTGAGGGGGAATG |
| miR166a-d,f_F | ACACTCCAGCTGGGTCGGACCAGGCTTCA |
| miR528_RT | CTCAACTGGTGTCGTGGAGTCGGCAATTCAGTTGAGCTCCTCTG |
| miR528_F | ACACTCCAGCTGGGTGGAAGGGGCATGCA |
| miR535_RT | CTCAACTGGTGTCGTGGAGTCGGCAATTCAGTTGAGGCGTGCTC |
| miR535_F | ACACTCCAGCTGGGTGACAACGAGAGAGA |
| miR812g,h,i_RT | CTCAACTGGTGTCGTGGAGTCGGCAATTCAGTTGAGTGTCCAAC |
| miR812g,h,i_F | ACACTCCAGCTGGGAAGACGGATGATTAAAGT |
| miR820a,b,c-5p.2_RT: | CTCAACTGGTGTCGTGGAGTCGGCAATTCAGTTGAGCTCCTGGT |
| miR820a,b,c-5p.2_F: | ACACTCCAGCTGGGTCGGCCTCGTGGATGGAC |
| miR1862d_RT | CTCAACTGGTGTCGTGGAGTCGGCAATTCAGTTGAGCGTCCCAA |
| miR1862d_F | ACACTCCAGCTGGGACTAGGTTTGTTTATTTT |
| miR1868_RT: | CTCAACTGGTGTCGTGGAGTCGGCAATTCAGTTGAGTGGCTGCT |
| miR1868_F: | ACACTCCAGCTGGGTCACGGAAAACGAGGGAG |
| miR408*_RT | CTCAACTGGTGTCGTGGAGTCGGCAATTCAGTTGAGCCATGCTC |
| miR408*_F | ACACTCCAGCTGGGCAGGGATGAGGCAGA |
| Universal primer | TGGTGTCGTGGAGTCG |
| action_F | GGAAGTACAGTGTCTGGATTGGAG |
| action_R | TCTTGGCTTAGCATTCTTGGGT |
| **Primers for 5'-RACE** | |
| BAK1 (Os06g16330) outer | ACTGCATCTCACTTACAATCAGTCATAT |
| BAK1 (Os06g16330) inner | GTCCTTGTCCTCTCCTCTTGTGTTTG |
| SBD (Os01g63810) outer | GAAAATATCCCATCTGCGTCACCAATCTC |
| SBD (Os01g63810) inner | CTTCGCCTCGTCCCAGTCCTCGCAG |
