# Supplementary material for: Characterization and Expression Patterns of microRNAs Involved in Rice Grain Filling
Source: PLoS One. 2013 Jan 24;8(1):e54148. doi: 10.1371/journal.pone.0054148 (PMC3554753; doi:10.1371/journal.pone.0054148)
Supplement: Table S2 — Predicted target fragments of miRNA*s from degradome data in starBase. (DOCX) [file pone.0054148.s005.docx]

**Table S2. Predicted target fragments of miRNA* from degradome data in starBase.**

| **miRNA*** | **Target** | **miRNA* squence** | **Degradome data** | **Cleavage tags** | | | **Penalty Score** | **Target Description** |
| --- | --- | --- | --- | --- | --- | --- | --- | --- |
|  |  |  |  | **GSM455938/rice seedling^a^** | **GSM455939/rice young panicle^a^** | **GSM476257/ young inflorescences (4~6cm)^a^** |  |  |
| miR1425* | LOC_Os08g09250 | CAGCAAGAACTGGATCTTAAT | ATTAAATCCAGTTGTTGCTG | 0 | 4 | 9 | 3 | glyoxalase family protein, putative, expressed |
| miR1433* | LOC_Os03g07880 | TAGCCAAGGATGATTTGCCTGT | ATGGCAAATCATCCTTGGCTTA | 305 | 6 | 58 | 3.5 | nuclear transcription factor Y subunit, putative, expressed |
| miR1433* | LOC_Os03g44540 | TAGCCAAGGATGATTTGCCTGT | AGGCAAATCATTCTTGGCTC | 0 | 25 | 97 | 4 | nuclear transcription factor Y subunit, putative, expressed |
| miR1884b* | LOC_Os01g11270 | AAAGTCAACGGTGTCATATATTTA | AAAATATTTGACACCGTTGACTTT | 0 | 0 | 1 | 2 | cytochrome P450, putative, expressed |

^a^ the rice degradome samples/sources.
