# Supplementary material for: Characterization and Expression Patterns of microRNAs Involved in Rice Grain Filling
Source: PLoS One. 2013 Jan 24;8(1):e54148. doi: 10.1371/journal.pone.0054148 (PMC3554753; doi:10.1371/journal.pone.0054148)
Supplement: Table S9 — Predicted target fragments of novel miRNAs from starBase. (DOCX) [file pone.0054148.s012.docx]

**Table S9. Predicted target fragments of novel miRNAs from starBase.**

| **miRNA** | **Target** | **miRNA squence** | **Degradome data** | **Cleavage tags** | | | **Penalty Score** | **Target Description** |
| --- | --- | --- | --- | --- | --- | --- | --- | --- |
|  |  |  |  | **GSM455938/rice seedling^a^** | **GSM455939/rice young panicle^a^** | **GSM476257/young inflorescences (4~6cm)^a^** |  |  |
| miRn17-5p | LOC_Os12g10710 | TCTTTCACATGGTATTAGAGCTG | GAGTATATACCATGTGAAAGA | 2 | 0 | 0 | 4 | NB-ARC domain containing protein, expressed |
| miRn21-5p | LOC_Os02g51790 | CAGGCAGAGCATGAAGAGCAT | AGCTCTTCATGCTCCGCCTC | 0 | 1 | 0 | 4 | ribosomal protein L29, putative, expressed |
| miRn24-3p | LOC_Os06g10890 | TTTGAACTTGATATTTGGTGG | CACTGATATCAAGTTCAAT | 1 | 1 | 0 | 4 | sterol carrier protein-2, putative, expressed |
| miRn24-3p | LOC_Os06g24730 | TTTGAACTTGATATTTGGTGG | CAATCTTTATCAAGTTCAAA | 0 | 1 | 1 | 4.5 | hydrolase, alpha/beta fold family domain containing protein, expressed |
| miRn33 -3p | LOC_Os03g52280 | TGAGATTGGTTTATTTTGGGA | TGCAAAATAAACCAATCTA | 0 | 0 | 2 | 4 | expressed protein |
| miRn45-5p | LOC_Os11g12810 | GCTGGAGTAGCTCAGATGGT | GCCATCTGAGCTAGTCAGC | 1 | 1 | 0 | 4.5 | sucrose-phosphate synthase, putative, expressed |

^a^ the rice degradome samples/sources.
